# Personalising the decision for prolonged dual antiplatelet therapy: development, validation and potential impact of prognostic models for cardiovascular events and bleeding in myocardial infarction survivors

**DOI:** 10.1093/eurheartj/ehw683

**Published:** 2017-02-27

**Authors:** Laura Pasea, Sheng-Chia Chung, Mar Pujades-Rodriguez, Alireza Moayyeri, Spiros Denaxas, Keith A.A. Fox, Lars Wallentin, Stuart J. Pocock, Adam Timmis, Amitava Banerjee, Riyaz Patel, Harry Hemingway

**Affiliations:** 1 The Farr Institute of Health Informatics Research, University College London, London, UK; 2 MRC Medical Bioinformatics Centre, Leeds Institute of Biomedical and Clinical Sciences, University of Leeds, UK; 3 Centre for Cardiovascular Science, University of Edinburgh and Royal Infirmary of Edinburgh, Edinburgh, UK; 4 Department of Medical Sciences Cardiology, Uppsala Clinical Research Centre, Uppsala University, Uppsala, Sweden; 5 Department of Medical Statistics, London School of Hygiene and Tropical Medicine, London, UK; 6 Bart’s Heart Centre, Barts and the London National Institute for Health Research Cardiovascular Biomedical Research Unit, London, UK

**Keywords:** Prognosis, Myocardial infarction, Bleeding

## Abstract

**Aims:**

The aim of this study is to develop models to aid the decision to prolong dual antiplatelet therapy (DAPT) that requires balancing an individual patient’s potential benefits and harms.

**Methods and results:**

Using population-based electronic health records (EHRs) (CALIBER, England, 2000–10), of patients evaluated 1 year after acute myocardial infarction (MI), we developed (*n* = 12 694 patients) and validated (*n* = 5613) prognostic models for cardiovascular (cardiovascular death, MI or stroke) events and three different bleeding endpoints. We applied trial effect estimates to determine potential benefits and harms of DAPT and the net clinical benefit of individuals. Prognostic models for cardiovascular events (c-index: 0.75 (95% CI: 0.74, 0.77)) and bleeding (c index 0.72 (95% CI: 0.67, 0.77)) were well calibrated: 3-year risk of cardiovascular events was 16.5% overall (5.2% in the lowest- and 46.7% in the highest-risk individuals), while for major bleeding, it was 1.7% (0.3% in the lowest- and 5.4% in the highest-risk patients). For every 10 000 patients treated per year, we estimated 249 (95% CI: 228, 269) cardiovascular events prevented and 134 (95% CI: 87, 181) major bleeding events caused in the highest-risk patients, and 28 (95% CI: 19, 37) cardiovascular events prevented and 9 (95% CI: 0, 20) major bleeding events caused in the lowest-risk patients. There was a net clinical benefit of prolonged DAPT in 63–99% patients depending on how benefits and harms were weighted.

**Conclusion:**

Prognostic models for cardiovascular events and bleeding using population-based EHRs may help to personalise decisions for prolonged DAPT 1-year following acute MI.

## Introduction

Among patients who survived a year since their last acute myocardial infarction (MI), subsequent major cardiovascular events, all-cause mortality and major bleeding risks are high.^1^^,^^2^ In unselected populations in USA, Sweden, England, and France, 20% of such patients experienced subsequent MI, stroke, or died during the following 3 years.^2^ Prolonged secondary prevention therapy in such patients is already recommended for four classes of drugs (statins, beta-blockers, ACE inhibitors, and aspirin). Recent trials^3^^,^^4^ examined addition of an extra antiplatelet. The PEGASUS-TIMI 54 trial^4^ found prolonged dual antiplatelet therapy (DAPT) using aspirin and ticagrelor compared with aspirin alone in patients 1–3 years since their last acute MI reduced the risk of cardiovascular death, stroke, or MI by 16% but increased major bleeding two-fold. In light of this evidence, 2015 European Society of Cardiology guidelines recommend prolonged DAPT may be ‘considered after careful assessment of ischaemic and bleeding risks’.^5^^,^^6^

How to make this ‘careful assessment’ is unclear. Risk prediction modelling has been proven invaluable in conditions such as atrial fibrillation where similar decisions on benefits and harm need to be weighed.^7^ Indeed, such models for bleeding and subsequent MI have been developed to guide use of bivalirudin,^8^ the choice of P2Y_12_ inhibitor,^9^ and duration of DAPT.^10^^,^^11^ An existing model intended to guide the duration of DAPT is based on patients undergoing drug eluting stent placement and selected into a trial and uses patient characteristics at the time of percutaneous coronary intervention.^11^ Currently, no prognostic models evaluate the long-term risks of bleeding and cardiovascular events using updated clinical information 1 year after acute MI, to support the key clinical decision on prolonging DAPT.

In this context, unselected populations are important to provide realistic estimates of long-term cardiovascular and bleeding risks; these estimates are often substantially higher than those observed in the placebo arms of trials.^12–14^ Linked electronic health records (EHRs) are ideal sources of data to derive such ‘real-life’ estimates of risks and harms, at scale. The CALIBER dataset,^15^ validated for cardiovascular prognostic research,^16–18^ is a 2 million person resource of linked primary–secondary and mortality data in England including 18 307 MI survivors.

Among these stable MI survivors, we sought to first, develop and validate prognostic models for major cardiovascular and bleeding events. We used prognostic factors present 1 year following acute MI and widely recorded as a part of guideline recommended care. Second, to demonstrate how predicted benefits and harms may aid treatment and clinical decisions, we applied PEGASUS-TIMI 54 trial^4^ relative risks of efficacy and safety to estimate potential numbers of cardiovascular events prevented and harms caused by prolonged DAPT, and net clinical benefits for individuals.

## Methods

The models were developed and validated in line with Transparent Reporting of a multivariable prediction model for Individual Prognosis Or Diagnosis (TRIPOD) guidelines^19^ (see Supplementary material online, *Table S1*).

### Linked EHRs

We used the CALIBER (ClinicAl research using LInked Bespoke and Electronic health Records) research platform, consisting of EHR linkages between primary care data (Clinical Practice Research Datalink (CPRD)), secondary care data (Hospital Episode Statistics), disease registry data (Myocardial Ischaemia National Audit Project), and cause-specific mortality (Office for National Statistics) in England.^15^ The 4% sample of England’s population in CPRD available for linkage is unselected, representative in terms of age, sex, and overall mortality.^20–22^ Furthermore, there is extensive evidence of risk factor and cardiovascular and non-cardiovascular disease endpoint validity in CALIBER.^16–18^^,^^23–27^ The study was approved by the Independent Scientific Advisory Committee of the Medicines and Health care products Regulatory Agency in the UK and the MINAP Academic Group.

### Study population

Patients in CALIBER alive 1 year after their last acute MI (i.e. their index acute MI) were included in the study. We studied patients from 2000 to 2010, before the introduction of ticagrelor, and when prolonged DAPT was rare. Follow-up started at 1 year after index acute MI, and patients were censored at the earliest date of the endpoints of interest, primary care practice transfer, death, or 5 years of follow-up. We evaluated prescriptions indicating prolonged clopidogrel use in follow up (defined in Supplementary material online, *Table S2*) in our cohort to ensure they were untreated with long-term DAPT i.e. the decision our models aimed to aid. Patients were split into model development and validation cohorts using a pre-specified geographical divide.^28^ The North of England has well-documented higher rates of cardiovascular mortality compared with the South.^29^ Based on the 10 administrative areas in the National Health Service, we chose 6 in the South for model development cohort and 4 in the North for model validation cohort.

### Potential prognostic factors

In model development, we considered *a priori* prognostic factors including demographics, behaviours, cardiovascular and non-cardiovascular medical history, medications, and clinical biomarkers. Each patient’s most recent biomarker records in the year from index acute MI to follow-up start were used. Medications were defined as having ≥1 prescription of a drug in the year prior to follow-up start. As a patient’s risk profile evolves with time (risk at index event and at one year may differ), we also analysed risk characteristics at the time of hospital discharge from index acute MI. Details on data sources and EHR phenotypes for prognostic factors are available at the CALIBER online portal (https://www.caliberresearch.org/portal/, 10 January 2017) and see Supplementary material online, *Table S2*.

### Endpoints

The primary endpoint relating to potential benefits of prolonged DAPT, a composite of cardiovascular death, MI, or ischemic or unspecified stroke, have validated phenotypes in CALIBER.^16^^,^^17^^,^^23^ For examining potential harms of prolonged DAPT, we evaluated three severe bleeding endpoints with differing incidence: (1) fatal or hospitalised bleeding; (2) CALIBER major bleeding (a composite of fatal bleeding, intracranial bleeding, hospitalised bleeding with length of stay exceeding 14 days and bleeding requiring transfusion); and (3) fatal or intracranial bleeding (see Supplementary material online, *Table S3*). We also evaluated models for all-cause mortality.

### Statistical analysis

#### Model development

We evaluated associations between endpoints and prognostic factors using proportional hazards models with Weibull baseline hazards. Univariable models were used to detect non-linear trends and inform inclusion of prognostic factors in multivariable models. Non-linear continuous prognostic factors were included in the model using restricted cubic splines. We chose the number of knots based on descriptive plots in the univariable analysis to provide a sufficient balance between capturing accurate shape and without overfitting.^30^ We used three knots (a sensitivity analysis using five knots resulted in very similar predictions). Proportional hazard assumptions were checked using residual and log(−log) plots. For non-normally distributed continuous prognostic factors, sensible functional form was obtained by log transformation. For parsimony, backwards selection methods were employed for constructing multivariable models, in which all prognostic factors were initially included, and removed stepwise if *P* > 0.1. Variance inflation factors were calculated to detect evidence of multicollinearity problems in the model selection process. Known important prognostic factors (age, gender, smoking status, index acute MI subtype, diabetes, history of MI prior to index acute MI, and stroke) were retained in all models. Missing values were multiply imputed using MICE (Multiple Imputation by Chained Equations).^31^ Thirty imputed sets were generated using all baseline covariates (demographics and behaviours, disease history, clinical biomarkers, and prescribed drugs) in the models.

#### Model validation

Models were validated using our geographically external North of England population. Unequal sized groups can minimise information loss when categorising compared with equal groupings,^32^ we, therefore, grouped patients into four risk groups (highest, high, low, and lowest) using cut-points at the 16th, 50th and 84th percentiles of the development cohort linear predictor.^33^ C-indexes estimated models discrimination. Model calibration was assessed visually by comparing plots of model expected events with validation cohort observed events, stratified by the risk group.

#### Model application

We applied relative risks for efficacy (cardiovascular death, stroke, or MI) and safety (TIMI major bleeding and fatal or intracranial bleeding) for ticagrelor 60 mg vs. placebo from the PEGASUS-TIMI 54 trial^4^ to the validation cohort stratified by predicted risk groups to estimate the potential events prevented and harms caused per 10 000 patients treated per year. We assume constant treatment effect across risk groups. We calculated net cardiovascular death, stroke, or MI and net CALIBER major bleeding risks with treatment for each individual in the validation cohort using the trial relative risk estimates. We evaluated predicted net benefit in patients (i.e. estimated cardiovascular risk decrease exceeds bleeding risk increase) under different benefit and harm weighting scenarios.^9^^,^^34^ Analyses were performed using R version 3.0.2.

## Results

### Baseline characteristics and overall event rates

The model development cohort consisted of 12 694 patients (mean age 70.1 years, 66.1% male) from 159 general practices, median follow-up of 3.1 years (range: 0–9.8) and 27% followed-up for at least 5 years (*Table **1* and see Supplementary material online, *Figure S1*). The validation cohort consisted of 5613 patients (mean age 69 years, 64.4% male) from 61 general practices. Overall, approximately 3% of patients had prolonged clopidogrel use in follow-up. Patient characteristics of the cohorts changed from acute MI discharge to our baseline of 1-year post-MI including increased heart failure and renal disease prevalence and changed smoking statuses (see Supplementary material online, *Table S4*). The cardiovascular and bleeding event rates from one-year post-MI in the development and validation cohorts are shown in Supplementary material online*, Figure S2*.

**Table 1:** Characteristics of population-based samples of patients at baseline defined as 1 year after their last acute MI

	Development cohort (*n*=12 694)	Validation cohort (*n*=5613)
Demographics and behaviours		
Age (years)	70.1 (12.7)	69.1 (12.8)
Women	33.9%	35.6%
Ethnicity		
Asian	2.0%	1.1%
Black	0.5%	0.1%
Other	17.0%	14.1%
White	80.4%	84.6%
Index of multiple deprivation (highest quartile- most deprived)	15.4%	30.3%
Smoking status		
Ex-smoker	49.3%	50.0%
Non-smoker	36.7%	34.2%
Smoker	14.0%	15.8%
Excess alcohol	10.8%	15.4%
Cardiovascular diseases		
Index acute MI subtype		
NSTEMI	32.1%	29.1%
STEMI	17.4%	16.4%
Unspecified	50.5%	54.5%
MI (prior to index acute MI)	34.7%	38.7%
Revascularisation (any)	43.5%	33.0%
Primary PCI for index acute MI	24.8%	18.8%
PCI at any time prior to 1 year post MI	39.4%	31.0%
CABG at any time to 1 year post MI	4.1%	2.0%
Stroke	6.9%	8.1%
Atrial fibrillation	18.0%	17.9%
Heart failure	23.5%	28.0%
Peripheral arterial disease	9.8%	13.1%
Renal disease	13.6%	14.8%
Recent hospitalisation for acute renal disease	1.3%	1.0%
Non-cardiovascular diseases		
Diabetes		
Type 1	1.2%	0.9%
Type 2	16.7%	17%
Unspecified	1.5%	1.7%
COPD	9.1%	12.8%
Recent hospitalisation for acute COPD	1.1%	2.2%
Liver disease	0.4%	0.5%
Non-metastatic cancer	14.4%	13.2%
Metastatic cancer	1.0%	1.2%
Dementia	1.3%	2.0%
Chronic anaemia	14.3%	17.9%
Peptic ulcer	7.3%	10.2%
Bleeding diatheses and coagulation disorders	1.1%	1.1%
Hospitalised bleeding	6.5%	8.2%
Treatments prescribed		
Aspirin	87.0%	86.2%
Clopidogrel	50.5%	47.8%
Prolonged clopidogrel, post-baseline	2.7%	3.4%
Oral anticoagulant	9.9%	9.5%
Statin	88.5%	88.6%
Anti-hypertensive	96.4%	96.0%
Biomarkers		
BMI (Continuous) (kg/m^2^)	27.8 (5.1)	27.7 (5.1)
BMI (Categorical)		
Underweight	1.5%	1.8%
Normal	28.7%	28.4%
Overweight	41.2%	42.2%
Obese	28.6%	27.7%
SBP (mmHg)	133 (18.6)	132 (18.4)
DBP (mmHg)	75.3 (10.4)	74.6 (10.1)
Haemoglobin (g/dl)	13.4 (1.6)	13.3 (1.6)
White blood cell count (10^9^/l)	7.60 (2.3)	7.68 (2.3)
Total cholesterol (mmol/l)	4.17 (1.0)	4.17 (1.0)
HDL cholesterol (mmol/l)	1.28 (0.4)	1.26 (0.4)
Creatinine (μmol/l) Median (IQR)	98 (84, 114)	99 (86, 117)
eGFR (ml/min)	65.5 (20.3)	64.7 (20.7)
eGFR < 60 ml/min	38.7%	41.2%

Values are mean  (SD) except where stated.

MI, myocardial infarction; STEMI, ST-elevation myocardial infarction; NSTEMI, non-ST-elevation myocardial infarction; PCI, Percutaneous coronary intervention; CABG, Coronary artery bypass graft; COPD, chronic obstructive pulmonary disease; BMI, body mass index; SBP, systolic blood pressure; DBP, diastolic blood pressure; HDL, high-density lipoprotein; eGFR, estimated glomerular filtration rate.

### Development of prognostic models

**Figure 1:**
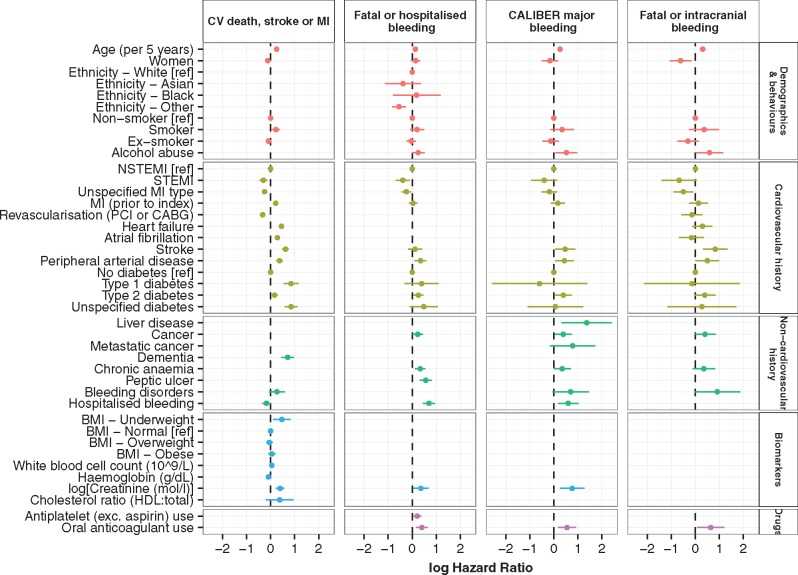
Prognostic factors (multivariable) for 5-year cardiovascular death stroke or MI, fatal or hospitalised bleeding, CALIBER major bleeding and fatal or intracranial bleeding endpoints. CV, cardiovascular; MI, myocardial infarction; ref, reference group; NSTEMI, non-ST-elevation myocardial infarction; STEMI, ST-elevation myocardial infarction; PCI, percutaneous coronary intervention; CABG, coronary artery bypass graft; BMI, body mass index; HDL, high-density lipoprotein; exc., excluding; log hazard ratios compared with [ref] group for categorical factors or per unit increase for continuous factors; for hazard ratios and 95% confidence intervals see Supplementary material online, *Table S4*. Systolic blood pressure was included in all models using restricted cubic splines (see Supplementary material online, *Table S5*).

In univariable (see Supplementary material online, *Figure S3*) and multivariable (*Figure **1* and see Supplementary material online, *Table S5*) modelling, we identified 20 prognostic factors for inclusion in our cardiovascular death, stroke, or MI model and 18 prognostic factors for inclusion in our three bleeding models. Prognostic factors were acceptable under the proportional hazards assumption (see Supplementary material online, *Figure S4*) and there was no evidence of problematic multicollinearity between the prognostic factors in the multivariable models (variance inflation factors < 3). Systolic blood pressure was modelled using restricted cubic splines due to a non-linear (U-shaped) relationship with the endpoints (see Supplementary material online, *Figure S5* and *Table S6*). The presence and direction of prognostic factor associations with cardiovascular events and bleeding outcomes were mostly concordant. The magnitude of prognostic factor associations (e.g. history of MI, stroke, diabetes) differed across endpoints.

### Validation of prognostic models

**Figure 2:**
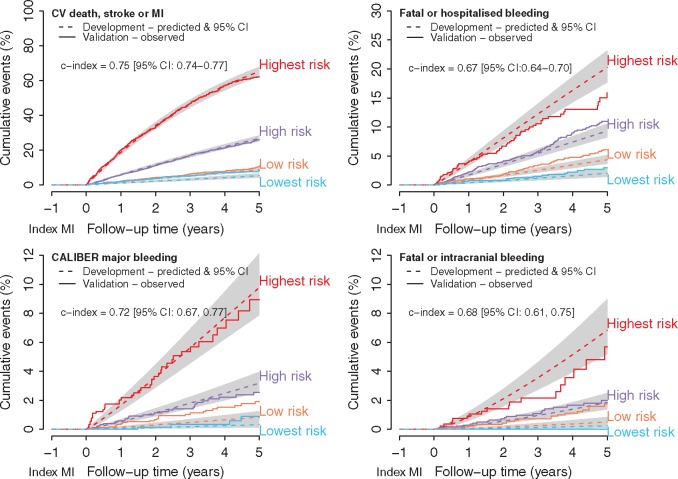
Geographical validation; calibration of cardiovascular and bleeding prognostic models by risk group. CV, cardiovascular; MI, myocardial infarction; CI, confidence interval.

For each endpoint, patients were divided into four risk groups (see Supplementary material online, *Table S7*). The models discriminated risk well (*Figure **2*), c-indexes of 0.75, 95% CI: 0.74, 0.77 for the composite of cardiovascular death, stroke or MI, CALIBER major bleeding (0.72, 95% CI: 0.67, 0.77), fatal or hospitalised bleeding (0.67, 95% CI: 0.64, 0.70), and fatal or intracranial bleeding (0.68, 95% CI: 0.61, 0.75). Better performance was observed for the all-cause mortality (0.81, 95% CI: 0.80, 0.82) and cardiovascular mortality (0.81, 95% CI: 0.80, 0.83) models. The models were highly calibrated (*Figure **2*), in particular, models for cardiovascular events performed satisfactorily across all risk groups.

### Potential absolute benefits and harms in risk groups

We observed higher event rates in our study cohort compared with the trial placebo group for cardiovascular death, stroke, or MI (16.5% vs. 9.04%) and major bleeding (CALIBER 1.7% vs. TIMI 1.26%) events. *Table **2* shows on an intention-to-treat basis in highest risk groups, for every 10 000 patients treated per year, 249 (95% CI: 228, 269) cardiovascular events may be prevented and 134 (95% CI: 87,181) major bleeding events may be caused, whereas in the lowest risk groups 28 (95% CI: 19, 37) cardiovascular events may be prevented and 9 (95% CI: 0, 20) major bleeding events may be caused. In the absence of risk stratification, we estimated 89 (95% CI: 83, 94) cardiovascular events prevented and 42 (95% CI: 32, 51) harmed patients per 10,000 treated per year. Fatal or intracranial bleeding occurred in 2.2% of high-risk patients and fatal or hospitalised bleeding occurred in 10.5% of high-risk patients.

**Table 2:** Estimated events prevented and harms caused per 10 000 patients treated per year with prolonged dual antiplatelet therapy by predicted risk groups compared with all risk groups combined and the PEGASUS-TIMI 54 trial population

	Unselected population					Trial population
	Risk groups as defined by prognostic models					
	in the validation cohort (*n*=5613)					
	Lowest	Low	High	Highest	All	PEGASUS-TIMI 54 placebo arm
Potential benefits						
Cardiovascular death, stroke, or MI						
3 year cumulative risk, % (95% CI)	5.2 (3.4, 6.9)	6.3 (5.0, 7.5)	17.1 (15.2, 19.0)	46.7 (42.7, 50.3)	16.5 (15.4, 17.6)	9.04
Events potentially prevented (95% CI) (ITT)	28 (19, 37)	34 (27, 41)	92 (81, 102)	249 (228, 269)	89 (83, 94)	42
Potential harms						
CALIBER major bleeding						
3-year cumulative risk, % (95% CI)	0.3 (0.0, 0.8)	1.0 (0.5, 1.5)	1.4 (0.8, 2.0)	5.4 (3.5, 7.2)	1.7 (1.3, 2.0)	1.26^a^ (ITT); 1.06^a^ (OT)
Harms potentially caused (95% CI) (ITT)	9 (0, 20)	26 (14, 39)	36 (21, 51)	134 (87, 181)	42 (32, 51)	31
Harms potentially caused (95% CI) (OT)	15 (0, 35)	46 (24, 68)	63 (36, 89)	236 (153, 318)	73 (56, 90)	47
Fatal bleeding or intracranial bleeding						
3- year cumulative risk, % (95% CI)	0	0.7 (0.3, 1.1)	1.1 (0.5, 1.6)	2.2 (0.9, 3.4)	0.9 (0.6, 1.2)	0.60
Harms potentially caused (95% CI) (OT)	–	5 (2, 8)	8 (4, 11)	15 (7, 23)	7 (5, 9)	4
Fatal or hospitalised bleeding						
3-year cumulative risk, % (95% CI)	1.4 (0.5, 2.3)	3.3 (2.4, 4.3)	5.8 (4.5, 7.0)	10.5 (8.0, 13.0)	4.9 (4.3, 5.6)	Not available^b^

*Note:* PEGASUS-TIMI 54 trial estimated relative risks [ticagrelor 60 mg vs. placebo; intention-to-treat  (ITT) and on treatment  (OT) estimates where available] for cardiovascular death, stroke, or MI [ITT: 0.84, main report], TIMI major bleeding [ITT: 1.75, appendix E; OT: 2.32, main report], fatal bleeding or intracranial bleeding [OT:1.20, main report] were used to calculate CV events potentially prevented and harms potentially caused per 10 000 treated per year.

a TIMI-major bleeding.

b No broad/composite bleeding endpoint reported in PEGASUS-TIMI 54.

### Potential net clinical benefits in individuals

**Figure 3:**
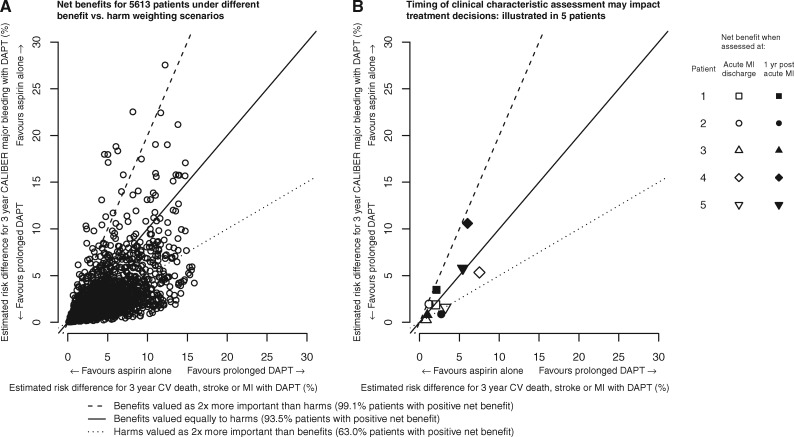
Net predicted risk for cardiovascular death, stroke, or MI and CALIBER major bleeding with prolonged dual antiplatelet therapy. MI, Myocardial infarction; CV, cardiovascular; DAPT, dual antiplatelet therapy. The straight lines correspond to different scenarios of how patients or clinicians may value (or weight) benefits and harms. All patients to the right of a line applicable to their values would be estimated to have a positive net benefit with prolonged DAPT. *Panel A:* Net benefit for 5613 patients under different benefit vs. harm weighting scenarios. *Panel B:* Timing of clinical characteristic assessment may impact treatment decisions shows the net benefits calculated using patient characteristics at discharge from acute MI and at 1 year post-acute MI for 5 ‘typical’ patients in the validation cohort (see Supplementary material online, *Table S4*).

We estimated positive net clinical benefit in 93.5% of patients when cardiovascular death, stroke, or MI events and CALIBER major bleeding were weighted equally, 63% if avoiding major bleeding was valued twice more than preventing cardiovascular events and 99.1% if preventing cardiovascular events was valued twice as high as avoiding major bleeding (*Figure **3*, *panel A*). We illustrate the importance of using clinical characteristics updated at 1 year after acute MI (*Figure **3*, *panel B*) compared with characteristics at acute MI discharge for five patients. For example, using characteristics at discharge Patient 5 would be considered suitable for prolonged DAPT under all weighting options (point ‘▽’ on the figure), whereas using updated information 1 year post-MI, this patient would only be considered suitable for prolonged DAPT if they valued potential benefits over potential harms of treatment (point ‘▼’ on the figure).

## Discussion

Using population-based linked EHRs, we developed and validated prognostic models providing personalised estimates of risks of major cardiovascular and bleeding events in patients 12 months after an acute MI. With trial relative risks, we estimated potential benefits and harms of prolonged DAPT across risk groups. In individuals, potential net clinical benefit was observed for the majority of patients, even when avoiding bleeding is considered twice as important as preventing cardiovascular events; however, the magnitude of benefit must also be considered.

### Potential cardiovascular event prevention

Our unselected study cohort experienced a much higher cardiovascular event rate than the PEGASUS-TIMI 54 trial placebo group despite the trial inclusion criteria enriching for high risk.^35^ Thus, the potential absolute benefit of prolonged DAPT may be greater than reported in the trial. Importantly, potential benefits are comparable when the unselected real-world study population reported here is restricted to those meeting the trial inclusion and exclusion criteria (89 vs. 101 events potentially prevented per 10 000 treated per year, respectively).^35^ Our models widely separated the risk of events: a clinician may treat nine times (28/249) as many patients in the lowest-risk, compared with the highest-risk group, to prevent one cardiovascular event. Our models help clarify limitations of ignoring individual patient characteristics evaluated during the stable phase post-acute MI.

### Potential bleeding harms

In balancing potential harms from prolonged DAPT, we show the importance of considering different bleeding endpoints. While all bleedings we studied may be considered serious (they all required hospitalisation), their event rates and potential harms vary significantly. We sought to approximate the PEGASUS-TIMI 54 trial primary safety endpoint, TIMI major bleeding, and successfully defined fatal bleeding, intracranial bleeding and transfusions, but not, in currently available EHR, acute haemoglobin change. Nonetheless, incidence of CALIBER major bleeding was comparable with TIMI major bleeding in the trial placebo arm.

### Balancing potential benefits and harms in individuals

Patients and clinicians differ in how they value different benefits and harms, and we derived net clinical benefit estimates under different weighting scenarios. These can inform patient counselling and patient–doctor discussions tailored to patients risk covariates. Cost-effectiveness considerations are additionally important in determining the magnitude of net clinical benefit a given health system is willing to pay for.^36^^,^^37^

### Need for multivariable risk prediction

The importance of tailoring risk to multiple patient characteristics, as opposed to single risk factors (e.g. age, diabetes, history of MI and renal disease)^38^ is illustrated in Supplementary material online, *Figure S6*. In each case, the risk distribution largely overlaps among people with and without simple binary prognostic factors. Furthermore, while simple, point-based scores for predicting bleeding risk prove valuable for other diseases,^39^^,^^40^ they are unlikely to be useful for this population. For example, we found HASBLED^7^ performed poorly in our stable post-MI cohort (c-index 0.57). It is well-known categorising clinical information loses predictive value and systolic blood pressure has non-linear U-shaped associations with endpoints (see Supplementary material online, *Figure S5* and *Table S6*)^41^ consistent with lower blood pressure reflecting impaired left ventricular function and therefore higher risk. Nonetheless in practice some simplification of our models will occur as absolute contraindications are established (e.g. although we adjusted for history of bleeding in our models, patients who bleed in the first year should be excluded from the prolonged DAPT treatment option).

### Application in clinical practice

We demonstrate marked changes in the year following acute MI in the prevalence of major prognostic factors, including heart failure, renal disease and smoking, with consequent changes in net benefits. Good clinical practice dictates thorough evaluation of patients at the time of decision-making including up-to-date medical history and biomarkers. Our models can be readily implemented in health systems with EHR.^42^ We present a web-based tool providing personalised risk predictions at http://www.caliberresearch.org/prolonged_dapt_benefits_harms_risks (10 January 2017).

### Methodological strengths

Few, if any, previous studies evaluated prognostic relevance of clinical data available at the decision point of surviving 1 year following an acute MI. Previous studies focused on factors measured in the acute hospitalised phase. Unlike voluntary disease registries or trial populations which may not reflect the entire population at risk, CALIBER is population based. Therefore, estimates of risk obtained are likely to be representative of those observed in usual clinical practice. Routinely collected clinical information readily available in EHR enabled estimation of changing net clinical benefit of prolonged DAPT during patient journeys. We have shown previously extensive evidence of the diagnostic and prognostic validity of the EHR sources in studies of acute myocardial infarction, stroke and bleeding endpoints.^14^^,^^16^^,^^17^^,^^23^

### Limitations

Our study has important limitations. First, the information that is recorded as part of usual clinical practice is unlikely to have the same precision as that recorded as part of standardised research protocols. If the quality of information recording is low, then this will diminish the ability of the models to discriminate risk. Second, current large-scale population-based EHR data lack information on left ventricular function, number of diseased vessels, coronary stent type, and diameter. However it is not known if such factors remain prognostically relevant when included in models with updated clinical information at 1-year post-MI. There is extensive evidence that stent type (bare metal, drug eluting, and bioabsorbable) is an important predictor of prognosis from the time of acute myocardial infarction, and this study was unable to evaluate whether, in the stable phase 12 months after acute myocardial infarction, at a time, when we demonstrate that clinical factors have changed, stent characteristics continue to provide incremental discrimination of risk. Previous nationwide studies of angiographic findings suggest only a modest predictor of subsequent events in stable patients.^43^ Furthermore, we did not validate our models in different health systems. However, a study of 140 000 unselected stable post-MI patients found similar rates of cardiovascular and bleeding events in England, France, Sweden, and USA^2^ and the effects of multiple prognostic factors reported in the present study were consistent across these four countries, suggesting potential geographic transportability of our models. We used multiple imputations where appropriate and our complete-case sensitivity analysis showed no important difference to our presented models. Multiply imputing MI type for those unspecified did not show any difference to the ST-elevation MI vs. non ST-elevation MI contrast presented in our multivariable models.

### Future research

We propose three major avenues for future research. First, further validation studies are recommended in populations differing in background, treatment strategies, and event rates to ensure generalisability.^43^ We selected a study period to provide a relatively pure population, prior to the UK approval of ticagrelor and widespread occurrences of prolonged DAPT. Further research is required to test our models in more recent cohorts with a mix of clopidogrel, ticagrelor, and prasugrel users and risk prediction models may be required for other harms (e.g. dyspnoea in patients taking ticagrelor). Second, as with any prognostic model the question arises of what is their impact on clinical outcomes and costs^36^^,^^37^ when implemented in clinical care.^44^ This would involve developing clinical decision support systems using prognostic models, and evaluation through a cluster randomised controlled trial. Third, there is a need to identify biomarkers which distinguish bleeding from cardiovascular event risks and may further aid decision-making.

## Conclusion

Using population-based EHRs, we developed and validated prognostic models for benefits and different bleeding harms, relevant to the decision to prolong DAPT in patients stable 1 year after an acute MI. Personalised treatment decisions, based on individual patient risk profiles, can inform decision-making.

## Funding

AstraZeneca, the Medical Research Council Prognosis Research Strategy (PROGRESS) Partnership (HH, Grant G0902393/99558), Medical Research Council Population Health Scientist Fellowship (S-CC: Grant MR/M015084/1), and by awards to establish the Farr Institute of Health Informatics Research, London, from the Medical Research Council, Arthritis Research UK, British Heart Foundation, Cancer Research UK, Chief Scientist Office, Economic and Social Research Council, Engineering and Physical Sciences Research Council, NIHR, National Institute for Social Care and Health Research, and Wellcome Trust (LP, S-CC, MP). The views expressed in this paper do not necessarily represent the views of the funding bodies. L.P. had full access to the data and take(s) responsibility for the integrity of the data and the accuracy of the data analysis. All authors had final responsibility for the decision to submit for publication. H.H. received funding from AstraZeneca to carry out this research project.

**Conflict of interest**: The sponsors did not have access to the data used and had no role in data collection, data curation, or analysis. The sponsors did comment on manuscript drafts but final decisions for analysis and publication were the responsibility of the authors.

## Supplementary Material

Supplementary Data

## References

[1] Jernberg T, Hasvold P, Henriksson M, Hjelm H, Thuresson M, Janzon M. Cardiovascular risk in post-myocardial infarction patients: nationwide real world data demonstrate the importance of a long-term perspective. Eur Heart J 2015;36:1163–1170. 25586123 10.1093/eurheartj/ehu505

[2] Rapsomaniki E, Thuresson M, Yang E, Blin P, Hunt P, Chung S-C, Stogiannis D, Pujades-Rodriguez M, Timmis A, Denaxas S, Danchin N, Stokes M, Thomas-Delecourt F, Emmas C, Hasvold P, Jennings E, Johansson S, Cohen DJ, Jernberg T, Moore N, Janzon M, Hemingway H. Using big data from health records from four countries to evaluate chronic disease outcomes: a study in 114 364 patients after myocardial infarction. Eur Heart J Qual Care Clin Outcomes 2016; http://dx.doi/10.1093/ehjqcco/qcw004. 10.1093/ehjqcco/qcw004 PMC5815620 29474617

[3] Mauri L, Kereiakes DJ, Yeh RW, Driscoll-Shempp P, Cutlip DE, Steg PG, Normand SL, Braunwald E, Wiviott SD, Cohen DJ, Holmes DR Jr., Krucoff MW, Hermiller J, Dauerman HL, Simon DI, Kandzari DE, Garratt KN, Lee DP, Pow TK, Ver Lee P, Rinaldi MJ, Massaro JM. Twelve or 30 months of dual antiplatelet therapy after drug-eluting stents. N Engl J Med 2014;371:2155–2166. 25399658 10.1056/NEJMoa1409312 PMC4481318

[4] Bonaca MP, Bhatt DL, Cohen M, Steg PG, Storey RF, Jensen EC, Magnani G, Bansilal S, Fish MP, Im K, Bengtsson O, Oude Ophuis T, Budaj A, Theroux P, Ruda M, Hamm C, Goto S, Spinar J, Nicolau JC, Kiss RG, Murphy SA, Wiviott SD, Held P, Braunwald E, Sabatine MS. Long-term use of ticagrelor in patients with prior myocardial infarction. N Engl J Med 2015;372:1791–1800. 25773268 10.1056/NEJMoa1500857

[5] Roffi M, Patrono C, Collet J-P, Mueller C, Valgimigli M, Andreotti F, Bax JJ, Borger MA, Brotons C, Chew DP, Gencer B, Hasenfuss G, Kjeldsen K, Lancellotti P, Landmesser U, Mehilli J, Mukherjee D, Storey RF, Windecker S. 2015 ESC Guidelines for the management of acute coronary syndromes in patients presenting without persistent ST-segment elevation. Eur Heart J 2015;37:267–315.26320110

[6] Keaney JF. Jr., Balancing the risks and benefits of dual platelet inhibition. N Engl J Med 2015;372:1854–1856. 25773507 10.1056/NEJMe1502137

[7] Pisters R, Lane DA, Nieuwlaat R, de Vos CB, Crijns HJ, Lip GY. A novel user-friendly score (HAS-BLED) to assess 1-year risk of major bleeding in patients with atrial fibrillation: the Euro Heart Survey. Chest 2010;138:1093–1100. 20299623 10.1378/chest.10-0134

[8] Pocock SJ, Mehran R, Clayton TC, Nikolsky E, Parise H, Fahy M, Lansky AJ, Bertrand ME, Lincoff AM, Moses JW, Ohman EM, White HD, Stone GW. Prognostic modeling of individual patient risk and mortality impact of ischemic and hemorrhagic complications: assessment from the Acute Catheterization and Urgent Intervention Triage Strategy trial. Circulation 2010;121:43–51. 20026777 10.1161/CIRCULATIONAHA.109.878017

[9] Salisbury AC, Wang K, Cohen DJ, Li Y, Jones PG, Spertus JA. Selecting antiplatelet therapy at the time of percutaneous intervention for an acute coronary syndrome: weighing the benefits and risks of prasugrel versus clopidogrel. Circ Cardiovasc Qual Outcomes 2013;6:27–34. 23212457 10.1161/CIRCOUTCOMES.112.965624

[10] Garg P, Galper BZ, Cohen DJ, Yeh RW, Mauri L. Balancing the risks of bleeding and stent thrombosis: a decision analytic model to compare durations of dual antiplatelet therapy after drug-eluting stents. Am Heart J 2015;169:222–233 e225. 25641531 10.1016/j.ahj.2014.11.002 PMC4407277

[11] Yeh RW, Secemsky E, Kereiakes DJ, Gershlick A, Cohen DJ, Spertus JA, Steg PG, Cutlip DE, Apruzzese PK, Massaro JM, Mauri L. Individualizing treatment duration of dual antiplatelet therapy after percutaneous coronary intervention: an analysis from the DAPT study. Circulation 2015;132:2267–2285.26644250

[12] Terkelsen CJ, Lassen JF, Norgaard BL, Gerdes JC, Jensen T, Gotzsche LB, Nielsen TT, Andersen HR. Mortality rates in patients with ST-elevation vs. non-ST-elevation acute myocardial infarction: observations from an unselected cohort. Eur Heart J 2005;26:18–26. 15615795 10.1093/eurheartj/ehi002

[13] Steg PG, Lopez-Sendon J, Lopez de Sa E, Goodman SG, Gore JM, Anderson FA Jr., Himbert D, Allegrone J, Van de Werf F. External validity of clinical trials in acute myocardial infarction. Arch Intern Med 2007;167:68–73. 17210880 10.1001/archinte.167.1.68

[14] Timmis A, Rapsomaniki E, Chung SC, Pujades-Rodriguez M, Moayyeri A, Stogiannis D, Shah AD, Pasea L, Denaxas S, Emmas C, Hemingway H. Prolonged dual antiplatelet therapy in stable coronary disease: comparative observational study of benefits and harms in unselected versus trial populations. BMJ 2016;353:i3163. 27334486 10.1136/bmj.i3163 PMC4916922

[15] Denaxas SC, George J, Herrett E, Shah AD, Kalra D, Hingorani AD, Kivimaki M, Timmis AD, Smeeth L, Hemingway H. Data resource profile: cardiovascular disease research using linked bespoke studies and electronic health records (CALIBER). Int J Epidemiol 2012;41:1625–1638. 23220717 10.1093/ije/dys188 PMC3535749

[16] Rapsomaniki E, Shah A, Perel P, Denaxas S, George J, Nicholas O, Udumyan R, Feder GS, Hingorani AD, Timmis A, Smeeth L, Hemingway H. Prognostic models for stable coronary artery disease based on electronic health record cohort of 102 023 patients. Eur Heart J 2014;35:844–852. 24353280 10.1093/eurheartj/eht533 PMC3971383

[17] Rapsomaniki E, Timmis A, George J, Pujades-Rodriguez M, Shah AD, Denaxas S, White IR, Caulfield MJ, Deanfield JE, Smeeth L, Williams B, Hingorani A, Hemingway H. Blood pressure and incidence of twelve cardiovascular diseases: lifetime risks, healthy life-years lost, and age-specific associations in 1.25 million people. Lancet 2014;383:1899–1911. 24881994 10.1016/S0140-6736(14)60685-1 PMC4042017

[18] George J, Rapsomaniki E, Pujades-Rodriguez M, Shah AD, Denaxas S, Herrett E, Smeeth L, Timmis A, Hemingway H. How does cardiovascular disease first present in women and men? Incidence of 12 cardiovascular diseases in a contemporary cohort of 1,937,360 people. Circulation 2015;CIRCULATIONAHA. 114.013797. 10.1161/CIRCULATIONAHA.114.013797 PMC4590518 26330414

[19] Collins GS, Reitsma JB, Altman DG, Moons KG. Transparent reporting of a multivariable prediction model for individual prognosis or diagnosis (TRIPOD): the TRIPOD statement. The TRIPOD Group. Circulation 2015;131:211–219. 25561516 10.1161/CIRCULATIONAHA.114.014508 PMC4297220

[20] Gallagher AM, Puri S, Staa TV. Linkage of the General Practice Research Database (GPRD) with other data sources. Pharmacoepidemiol Drug Saf 2011;20:230–364. 10.1002/pds.2206.

[21] Mathur R, Bhaskaran K, Chaturvedi N, Leon DA, vanStaa T, Grundy E, Smeeth L. Completeness and usability of ethnicity data in UK-based primary care and hospital databases. J Public Health (Oxf) 2014;36:684–692. 24323951 10.1093/pubmed/fdt116 PMC4245896

[22] Herrett E, Gallagher AM, Bhaskaran K, Forbes H, Mathur R, van Staa T, Smeeth L. Data resource profile: Clinical Practice Research Datalink (CPRD). Int J Epidemiol 2015;44:827–836. 26050254 10.1093/ije/dyv098 PMC4521131

[23] Herrett E, Shah AD, Boggon R, Denaxas S, Smeeth L, van Staa T, Timmis A, Hemingway H. Completeness and diagnostic validity of recording acute myocardial infarction events in primary care, hospital care, disease registry, and national mortality records: cohort study. BMJ 2013;346:f2350. 23692896 10.1136/bmj.f2350 PMC3898411

[24] Morley KI, Wallace J, Denaxas SC, Hunter RJ, Patel RS, Perel P, Shah AD, Timmis AD, Schilling RJ, Hemingway H. Defining disease phenotypes using national linked electronic health records: a case study of atrial fibrillation. PLoS ONE 2014;9:e110900. 25369203 10.1371/journal.pone.0110900 PMC4219705

[25] Pujades-Rodriguez M, George J, Shah AD, Rapsomaniki E, Denaxas S, West R, Smeeth L, Timmis A, Hemingway H. Heterogeneous associations between smoking and a wide range of initial presentations of cardiovascular disease in 1 937 360 people in England: lifetime risks and implications for risk prediction. Int J Epidemiol 2015;44:129–141. 25416721 10.1093/ije/dyu218 PMC4339760

[26] Pujades-Rodriguez M, Timmis A, Stogiannis D, Rapsomaniki E, Denaxas S, Shah A, Feder G, Kivimaki M, Hemingway H. Socioeconomic deprivation and the incidence of 12 cardiovascular diseases in 1.9 million women and men: implications for risk prediction and prevention. PLoS ONE 2014;9:e104671. 25144739 10.1371/journal.pone.0104671 PMC4140710

[27] Shah AD, Langenberg C, Rapsomaniki E, Denaxas S, Pujades-Rodriguez M, Gale CP, Deanfield J, Smeeth L, Timmis A, Hemingway H. Type 2 diabetes and incidence of cardiovascular diseases: a cohort study in 1.9 million people. Lancet Diabetes Endocrinol 2015;3:105–113. 25466521 10.1016/S2213-8587(14)70219-0 PMC4303913

[28] Moons KGM, Kengne AP, Grobbee DE, Royston P, Vergouwe Y, Altman DG, Woodward M. Risk prediction models: II. External validation, model updating, and impact assessment. Heart 2012;98:691–698. 22397946 10.1136/heartjnl-2011-301247

[29] Muller-Nordhorn J, Binting S, Roll S, Willich SN. An update on regional variation in cardiovascular mortality within Europe. Eur Heart J 2008;29:1316–1326. 18256043 10.1093/eurheartj/ehm604

[30] Collins GS, Ogundimu EO, Cook JA, Manach YL, Altman DG. Quantifying the impact of different approaches for handling continuous predictors on the performance of a prognostic model. Stat Med 2016;10.1002/sim.6986:n/a-n/a. 10.1002/sim.6986 PMC5026162 27193918

[31] Buuren S, Groothuis-Oudshoorn K. MICE: multivariate imputation by chained equations in R. J Stat Softw 2011;45.

[32] Cox DR. Note on Grouping. J Am Stat Assoc 1957;52:543–547.

[33] Royston P, Altman DG. External validation of a Cox prognostic model: principles and methods. BMC Med Res Methodol 2013;13:33. 23496923 10.1186/1471-2288-13-33 PMC3667097

[34] Pocock SJ, Stone GW, Mehran R, Clayton TC. Individualizing treatment choices using quantitative methods. Am Heart J 2014;168:607–610. 25440787 10.1016/j.ahj.2014.08.003

[35] Rapsomaniki E, Stogiannis D, Emmas C, Chung S, Pasea L, Denaxas S, Shah A, Pujades-Rodriguez M, Timmis A, Hemingway H, Health outcomes in patients with stable coronary artery disease following myocardial infarction; construction of a PEGASUS-TIMI-54 like population in UK linked electronic health records (Abstract). ESC 2014;35:173–512; 10.1093/eurheartj/ehu323.

[36] Walker SM, Asaria M, Manca A, Palmer SJ, Gale C, Shah AD, Abrams K, Crowther M, Timmis A, Hemingway H, Sculpher M. Long term health care use and costs in patients with stable coronary artery disease: a population based cohort using linked electronic health records (CALIBER). Eur Heart J Qual Care Clin Outcomes 2016; 10.1093/ehjqcco/qcw003. PMC4816202 27042338

[37] Asaria M, Walker SM, Palmer SJ, Gale C, Shah AD, Abrams K, Crowther M, Manca A, Timmis A, Hemingway H, Sculpher M. Using electronic health records to predict costs and outcomes in stable coronary artery disease. Heart 2016; 10.1136/heartjnl-2015-308850. PMC4849559 26864674

[38] Bonaca MP, Bhatt DL, Braunwald E, Cohen M, Steg PG, Storey RF, Held P, Jensen EC, Sabatine MS. Design and rationale for the Prevention of Cardiovascular Events in Patients With Prior Heart Attack Using Ticagrelor Compared to Placebo on a Background of Aspirin-Thrombolysis in Myocardial Infarction 54 (PEGASUS-TIMI 54) trial. Am Heart J 2014;167:437–U444. 24655690 10.1016/j.ahj.2013.12.020

[39] Lip GY, Nieuwlaat R, Pisters R, Lane DA, Crijns HJ. Refining clinical risk stratification for predicting stroke and thromboembolism in atrial fibrillation using a novel risk factor-based approach: the euro heart survey on atrial fibrillation. Chest 2010;137:263–272. 19762550 10.1378/chest.09-1584

[40] Melgaard L, Gorst-Rasmussen A, Lane DA, Rasmussen L, Larsen T, Lip GH. Assessment of the cha2ds2-vasc score in predicting ischemic stroke, thromboembolism, and death in patients with heart failure with and without atrial fibrillation. JAMA 2015;314:1030–1038. 26318604 10.1001/jama.2015.10725

[41] Stenestrand U, Wijkman M, Fredrikson M, Nystrom FH. Association between admission supine systolic blood pressure and 1-year mortality in patients admitted to the intensive care unit for acute chest pain. JAMA 2010;303:1167–1172. 20332402 10.1001/jama.2010.314

[42] Hippisley-Cox J, Coupland C, Vinogradova Y, Robson J, Minhas R, Sheikh A, Brindle P. Predicting cardiovascular risk in England and Wales: prospective derivation and validation of QRISK2. BMJ 2008;336:1475–1482. 18573856 10.1136/bmj.39609.449676.25 PMC2440904

[43] Henriksson M, Palmer S, Chen R, Damant J, Fitzpatrick NK, Abrams K, Hingorani AD, Stenestrand U, Janzon M, Feder G, Keogh B, Shipley MJ, Kaski JC, Timmis A, Sculpher M, Hemingway H. Assessing the cost effectiveness of using prognostic biomarkers with decision models: case study in prioritising patients waiting for coronary artery surgery. BMJ 2010;340:b5606. 20085988 10.1136/bmj.b5606 PMC2808469

[44] Steyerberg EW, Moons KG, van der Windt DA, Hayden JA, Perel P, Schroter S, Riley RD, Hemingway H, Altman DG. Prognosis Research Strategy (PROGRESS) 3: prognostic model research. PLoS Med 2013;10:e1001381. 23393430 10.1371/journal.pmed.1001381 PMC3564751

