# Supplementary material for: Personalising the decision for prolonged dual antiplatelet therapy: development, validation and potential impact of prognostic models for cardiovascular events and bleeding in myocardial infarction survivors
Source: Eur Heart J. 2017 Feb 27;38(14):1048–55. doi: 10.1093/eurheartj/ehw683 (PMC5400049; doi:10.1093/eurheartj/ehw683)
Supplement: Supplementary Data [file ehw683_supp.docx]

**SUPPLEMENTARY APPENDIX**

The contents of the supplementary appendix are aimed to provide a deeper understanding towards the analyses performed for this study. It enables replication of the models, through outcome and prognostic factor definitions and exact specification of the developed models.

**Contents**

| eTable 1 | Completed TRIPOD checklist |
| --- | --- |
| eTable 2 | Timing and sources of prognostic factors |
| eTable 3 | Defining three bleeding endpoints using codes in linked electronic health records |
| eFigure 1 | Study population flow diagram, endpoints and 3 & 5 year event rates |
| eTable 4 | Patient characteristics at index acute MI discharge and at 1 year post-index acute MI in the development (n=12,694) and validation (n=5,613) cohorts |
| eFigure 2 | Comparison of all-cause mortality, cardiovascular and bleeding events in patents included in the development (n=12694) and validation (n=5,613) cohorts |
| eFigure 3 | Univariable effects of prognostic factors on 5 year all-cause mortality, cardiovascular and bleeding endpoints |
| eFigure 4 | Univariable proportional hazards assumption checks for the CALIBER major bleeding outcome |
| eTable 5 | Multivariable model prognostic hazard ratios and 95% confidence intervals for all-cause mortality, cardiovascular and bleeding endpoints |
| eFigure 5 | U-shaped association of systolic blood pressure and 5 year cardiovascular death, stroke or MI events [n=12,694, events=1,913] |
| eTable 6 | Linear predictor functions for systolic blood pressure in the multivariable models |
| eTable 7 | Percentages of patients in the 4 risk groups from applying the 16%, 50% and 84% cut-points of the linear predictors calculated with the development cohort for each endpoint |
| eFigure 6 | Overlap of 3 year predicted risks based on multivariable models in those with and without categorical risk factors: age≥ 65, diabetes, history of MI and renal disease (used to define high risk in PEGASUS-TIMI 54) |
| eFigure 7 | CALIBER DAPT online risk calculator illustrating risks of 3 year cardiovascular and bleeding events according to input characteristics with and without prolonged DAPT |

**eTable 1: Completed TRIPOD checklist**

| **Section/Topic** | **Item** |  | **Checklist Item** | **Page** |
| --- | --- | --- | --- | --- |
| Title and abstract | | | | |
| Title | 1 | D;V | Identify the study as developing and/or validating a multivariable prediction model, the target population, and the outcome to be predicted. | 1 |
| Abstract | 2 | D;V | Provide a summary of objectives, study design, setting, participants, sample size, predictors, outcome, statistical analysis, results, and conclusions. | 2 |
| Introduction | | | | |
| Background and objectives | 3a | D;V | Explain the medical context (including whether diagnostic or prognostic) and rationale for developing or validating the multivariable prediction model, including references to existing models. | 3 |
|  | 3b | D;V | Specify the objectives, including whether the study describes the development or validation of the model or both. | 4 |
| Methods | | | | |
| Source of data | 4a | D;V | Describe the study design or source of data (e.g., randomized trial, cohort, or registry data), separately for the development and validation data sets, if applicable. | 5 |
|  | 4b | D;V | Specify the key study dates, including start of accrual; end of accrual; and, if applicable, end of follow-up. | 5 |
| Participants | 5a | D;V | Specify key elements of the study setting (e.g., primary care, secondary care, general population) including number and location of centres. | 5 |
|  | 5b | D;V | Describe eligibility criteria for participants. | 5 |
|  | 5c | D;V | Give details of treatments received, if relevant. | n/a |
| Outcome | 6a | D;V | Clearly define the outcome that is predicted by the prediction model, including how and when assessed. | 6; eTable 3 |
|  | 6b | D;V | Report any actions to blind assessment of the outcome to be predicted. | n/a |
| Predictors | 7a | D;V | Clearly define all predictors used in developing the multivariable prediction model, including how and when they were measured. | 6; eTable 2 |
|  | 7b | D;V | Report any actions to blind assessment of predictors for the outcome and other predictors. | n/a |
| Sample size | 8 | D;V | Explain how the study size was arrived at. | 6 |
| Missing data | 9 | D;V | Describe how missing data were handled (e.g., complete-case analysis, single imputation, multiple imputation) with details of any imputation method. | 7 |
| Statistical analysis methods | 10a | D | Describe how predictors were handled in the analyses. | 6-7 |
|  | 10b | D | Specify type of model, all model-building procedures (including any predictor selection), and method for internal validation. | 6-7 |
|  | 10c | V | For validation, describe how the predictions were calculated. | 7 |
|  | 10d | D;V | Specify all measures used to assess model performance and, if relevant, to compare multiple models. | 7 |
|  | 10e | V | Describe any model updating (e.g., recalibration) arising from the validation, if done. | n/a |
| Risk groups | 11 | D;V | Provide details on how risk groups were created, if done. | 7 |
| Development vs. validation | 12 | V | For validation, identify any differences from the development data in setting, eligibility criteria, outcome, and predictors. | 5,Table 1; eFigure 2 |
| Results | | | | |
| Participants | 13a | D;V | Describe the flow of participants through the study, including the number of participants with and without the outcome and, if applicable, a summary of the follow-up time. A diagram may be helpful. | eFigure 1 |
|  | 13b | D;V | Describe the characteristics of the participants (basic demographics, clinical features, available predictors), including the number of participants with missing data for predictors and outcome. | Table 1 |
|  | 13c | V | For validation, show a comparison with the development data of the distribution of important variables (demographics, predictors and outcome). | Table 1; eTable 4; eFigure 5 |
| Model development | 14a | D | Specify the number of participants and outcome events in each analysis. | eFigure 1 |
|  | 14b | D | If done, report the unadjusted association between each candidate predictor and outcome. | eFigure 3 |
| Model specification | 15a | D | Present the full prediction model to allow predictions for individuals (i.e., all regression coefficients, and model intercept or baseline survival at a given time point). | eTable 5-6 |
|  | 15b | D | Explain how to use the prediction model. | 11 |
| Model performance | 16 | D;V | Report performance measures (with CIs) for the prediction model. | 8-9 |
| Model-updating | 17 | V | If done, report the results from any model updating (i.e., model specification, model performance). | n/a |
| Discussion | | | | |
| Limitations | 18 | D;V | Discuss any limitations of the study (such as nonrepresentative sample, few events per predictor, missing data). | 12 |
| Interpretation | 19a | V | For validation, discuss the results with reference to performance in the development data, and any other validation data. | 10-11 |
|  | 19b | D;V | Give an overall interpretation of the results, considering objectives, limitations, results from similar studies, and other relevant evidence. | 10-11; Table 2 |
| Implications | 20 | D;V | Discuss the potential clinical use of the model and implications for future research. | 11-12 |
| Other information | | | | |
| Supplementary information | 21 | D;V | Provide information about the availability of supplementary resources, such as study protocol, Web calculator, and data sets. | Web calculator available with publication; eFigure 7 |
| Funding | 22 | D;V | Give the source of funding and the role of the funders for the present study. | 14 |

*Items relevant only to the development of a prediction model are denoted by D, items relating solely to a validation of a prediction model are denoted by V, and items relating to both are denoted D;V.

**eTable 2: Timing and sources of prognostic factors**

| **Prognostic Factor** | **Definitions** | **Timing of measure** |
| --- | --- | --- |
| Age (years) |  | At cohort entry |
| Gender |  | - |
| Smoking status | <https://www.caliberresearch.org/portal/show/smoking_status_composite> | Most recent value recorded in the year prior to cohort entry |
| Excess alcohol | https://www.caliberresearch.org/portal/show/alcohol_drinker_composite | At any time prior to cohort entry |
| Index of multiple deprivation |  |  |
| Ethnicity |  | - |
| Index MI subtype | <https://www.caliberresearch.org/portal/show/phenotype_mi> | Index MI |
| Diabetes | <https://www.caliberresearch.org/portal/show/phenotype_diabetes> | At any time prior to cohort entry |
| History of MI (prior to index MI) | <https://www.caliberresearch.org/portal/show/phenotype_mi> | At any time prior to index MI |
| History of stroke | <https://www.caliberresearch.org/portal/show/ischaemic_stroke_gprd>  <https://www.caliberresearch.org/portal/show/ischaemic_stroke_hes>  <https://www.caliberresearch.org/portal/show/stroke_nos_gprd>  <https://www.caliberresearch.org/portal/show/stroke_nos_hes> | At any time prior to cohort entry |
| Previous revascularisation | <https://www.caliberresearch.org/portal/show/pci_gprd>  <https://www.caliberresearch.org/portal/show/pci_opcs>  <https://www.caliberresearch.org/portal/show/cabg_gprd>  <https://www.caliberresearch.org/portal/show/cabg_opcs> | At any time prior to cohort entry |
| History of atrial fibrillation | <https://www.caliberresearch.org/portal/show/phenotype_af> | At any time prior to cohort entry |
| History of heart failure | <https://www.caliberresearch.org/portal/show/phenotype_hf> | At any time prior to cohort entry |
| History of peripheral arterial disease | <https://www.caliberresearch.org/portal/show/phenotype_pad> | At any time prior to cohort entry |
| Chronic anaemia | <https://www.caliberresearch.org/portal/show/chronicanaemia_gprd>  <https://www.caliberresearch.org/portal/show/chronicanaemia_hes> | At any time prior to cohort entry |
| History of hospitalised bleed | **ICD-10**   \| I60 \| I61 \| I62 \| K250 \| K282 \| K284 \| K286 \| K290 \| \| --- \| --- \| --- \| --- \| --- \| --- \| --- \| --- \| \| K252 \| K254 \| K256 \| K260 \| K625 \| K920 \| K921 \| K922 \| \| K262 \| K264 \| K266 \| K270 \| P261 \| R040 \| R041 \| R048 \| \| K272 \| K274 \| K276 \| K280 \| R049 \| H356 \| H431 \| H450 \| | At any time prior to cohort entry |
| History of peptic ulcer | <https://www.caliberresearch.org/portal/show/pepticulcer_gprd>  <https://www.caliberresearch.org/portal/show/pepticulcer_hes> | At any time prior to cohort entry |
| Bleeding diatheses and coagulation disorders | **ICD-10 codes**   \| D66X \| D67X \| D680 \| D681 \| D691 \| D692 \| D693 \| D694 \| \| --- \| --- \| --- \| --- \| --- \| --- \| --- \| --- \| \| D682 \| D683 \| D684 \| D685 \| D695 \| D696 \| D698 \| D699 \| \| D686 \| D688 \| D689 \| D690 \|   **Read codes**   \| D304.00 \| D305.00 \| D307.00 \| D307y00 \| D307z00 \| Dyu3000 \| \| --- \| --- \| --- \| --- \| --- \| --- \| \| D310.00 \| D310z00 \| D311.00 \| D312.00 \| Dyu3100 \| D313.12 \| \| D313000 \| D313012 \| Dyu3200 \| D314.00 \| D314y00 \| D314z00 \| \| D31y.00 \| Dyu3300 \| D31X.00 \| D300.00 \| D300.12 \| D301.00 \| \| D301.12 \| D302.00 \| D302.11 \| D303.00 \| D30A.00 \| D30..00 \| \| D30z.00 \| 42P2.00 \| 42P2.11 \| D313.00 \| D313111 \| D313300 \| \| D313y00 \| D313z00 \| D313z11 \| D314100 \| D315.00 \|  \| | At any time prior to cohort entry |
| History of COPD | <https://www.caliberresearch.org/portal/show/copd_gprd>  <https://www.caliberresearch.org/portal/show/copd_hes> | At any time prior to cohort entry |
| Recent hospitalisation for acute COPD | <https://www.caliberresearch.org/portal/show/copd_hes> | In the year prior to cohort entry |
| Liver disease | <https://www.caliberresearch.org/portal/show/liver_charlson_gprd>  <https://www.caliberresearch.org/portal/show/liver_charlson_hes>  <https://www.caliberresearch.org/portal/show/cirrhosis_gprd>  <https://www.caliberresearch.org/portal/show/cirrhosis_hes>  <https://www.caliberresearch.org/portal/show/pbc_diag_gprd>  <https://www.caliberresearch.org/portal/show/pbc_diag_hes> | At any time prior to cohort entry |
| History of renal disease | [https://www.caliberresearch.org/portal/show/renal_gprd https://www.caliberresearch.org/portal/show/renal_hes](https://www.caliberresearch.org/portal/show/renal_hes) | At any time prior to cohort entry |
| Recent hospitalisation for acute renal disease | <https://www.caliberresearch.org/portal/show/renal_hes> | In the year prior to cohort entry |
| History of non-metastatic cancer | [https://www.caliberresearch.org/portal/show/cancer_gprd https://www.caliberresearch.org/portal/show/cancer_hes](https://www.caliberresearch.org/portal/show/cancer_gprd) | At any time prior to cohort entry |
| History of metastatic cancer | [https://www.caliberresearch.org/portal/show/cancer_gprd https://www.caliberresearch.org/portal/show/cancer_hes](https://www.caliberresearch.org/portal/show/cancer_gprd) | At any time prior to cohort entry |
| History of dementia | <https://www.caliberresearch.org/portal/show/dementia_gprd>  <https://www.caliberresearch.org/portal/show/dementia_hes> | At any time prior to cohort entry |
| BMI | <https://www.caliberresearch.org/portal/show/bmi> | Most recent value recorded in the year prior to cohort entry |
| SBP (mmHg) | <https://www.caliberresearch.org/portal/show/bp_gprd> |  |
| DBP (mmHg) | <https://www.caliberresearch.org/portal/show/bp_gprd> |  |
| Pulse rate (bpm) | <https://www.caliberresearch.org/portal/show/pulse_rate_gprd> |  |
| Haemoglobin (g/dL) | <https://www.caliberresearch.org/portal/show/haemoglobin_gprd> |  |
| Total cholesterol (mmol/L) | <https://www.caliberresearch.org/portal/show/total_chol_serum_gprd> |  |
| HDL cholesterol (mmol/L) | <https://www.caliberresearch.org/portal/show/HDL_serum_gprd> |  |
| Creatinine (mol/l) | <https://www.caliberresearch.org/portal/show/crea_gprd> |  |
| eGFR (ml/min) | <https://www.caliberresearch.org/portal/show/egfr_ckdepi_gprd> |  |

**eTable 3: Defining three bleeding endpoints using codes in linked electronic health records**

| **Endpoint** | **ICD-10 codes** |
| --- | --- |
| Fatal or hospitalised bleeding | In hospital admissions (HES) OR death registry(ONS):   \| I60 \| I61 \| I62 \| K250 \| K252 \| K254 \| K256 \| \| --- \| --- \| --- \| --- \| --- \| --- \| --- \| \| K260 \| K262 \| K264 \| K266 \| K270 \| K272 \| K274 \| \| K276 \| K280 \| K282 \| K284 \| K286 \| K290 \| K625 \| \| K920 \| K921 \| K922 \| P261 \| R040 \| R041 \| R048 \| \| R049 \| H356 \| H431 \| H450 \|  \|  \|  \| \|  \|  \|  \|  \|  \|  \|  \| |
| CALIBER major bleeding | In hospital admissions (HES):   \| I60 \| I61 \| I62 \| \| --- \| --- \| --- \|   In death registry (ONS):   \| I60 \| I61 \| I62 \| K250 \| K252 \| K254 \| K256 \| \| --- \| --- \| --- \| --- \| --- \| --- \| --- \| \| K260 \| K262 \| K264 \| K266 \| K270 \| K272 \| K274 \| \| K276 \| K280 \| K282 \| K284 \| K286 \| K290 \| K625 \| \| K920 \| K921 \| K922 \| P261 \| R040 \| R041 \| R048 \| \| R049 \| H356 \| H431 \| H450 \|  \|  \|  \|   **Or** all-cause mortality within 7 days of a hospital admission for any of the above codes  **Or** any of the above codes in hospital admissions with primary admission and hospitalisation >14days  **Or** any of the above codes in hospital admissions with a transfusion code in primary care (Read codes 7L14.00, 7L14000, 7L14100, 7L14300, 7L14311, 7L14y00, 7L14z00, TAy0.00, TB1y000, ZV58200) or hospital (OPCS codes X33, X331, X332, X333, X338, X339) within 30 days |
| Fatal or intracranial bleeding | In hospital admissions (HES):   \| I60 \| I61 \| I62 \| \| --- \| --- \| --- \|   In death registry(ONS):   \| I60 \| I61 \| I62 \| K250 \| K252 \| K254 \| K256 \| \| --- \| --- \| --- \| --- \| --- \| --- \| --- \| \| K260 \| K262 \| K264 \| K266 \| K270 \| K272 \| K274 \| \| K276 \| K280 \| K282 \| K284 \| K286 \| K290 \| K625 \| \| K920 \| K921 \| K922 \| P261 \| R040 \| R041 \| R048 \| \| R049 \| H356 \| H431 \| H450 \|  \|  \|  \|   **Or** all-cause mortality within 7 days of a hospital admission for any of the above codes |

**Note**: the prefix of the code can identify the bleeding location: I=Intracranial, K=Gastrointestinal, P=pulmonary, R=respiratory, H= Eye; Fatal or hospitalised bleeding included fatal bleeding or hospitalisation of any duration with bleeding as a primary or secondary reason for admission; CALIBER major bleeding included fatal or intracranial bleeding, bleeding as a primary cause of hospitalisation with length of stay > 14 days or bleedings requiring transfusion. Bleeding requiring transfusion were identified as bleedings with a relevant transfusion record in either primary or secondary care within 30 days following the bleeding; Fatal or intracranial bleeding included fatal bleeding or intracranial bleeding only. Bleedings were considered fatal if there was a bleeding code recorded as the underlying cause of death or if a patient died from any cause within 7 days of hospitalised bleeding;

**eFigure 1: Study population flow diagram, endpoints and 3 & 5 year event rates**

Prognostic model **validation** **population** (n= 5,613 in 61 general practices from East Midlands and North England)

Patients with an acute MI recorded in CALIBER 2000-2010

(n= 48,222)

Patients stable 1 year post-MI

(n=18,307)

Index acute MI subtype:

- STEMI (n= 3,127)
- NSTEMI (n= 5,712)
- Unclassified (n= 9,468)

Excluded (n= 29,915) due to:

- Fatal index acute MI or recurrent MI within 365 days (n=13,843)
- Died prior to 1 year/ transferred out of CPRD practice (n= 8,860)
- Less than 1 year at CPRD practice prior to index acute MI (n= 7,212)

Prognostic model **development** **population** (n= 12,694 in 159 general practices from South England, London, East England and West Midlands)

| **Endpoints from 1 year post MI** | **3 year events, n (%)** | **5 year events, n (%)** | |
| --- | --- | --- | --- |
| All-cause mortality | | |  |
|  | 1,943 (18.7) | 2,683 (30.1) | |
| CV death or ischaemic/ NOS stroke or MI | | |  |
|  | 1,469 (14.6) | 1,913 (22.4) | |
| CV death |  |  | |
|  | 1,020 (10.2) | 1,374 (16.5) | |
| Fatal or hospitalised bleeding | | |  |
|  | 408 (4.3) | 556 (7.3) | |
| CALIBER major bleeding | | |  |
|  | 141 (1.5) | 188 (2.5) | |
| Fatal bleeding or intracranial bleeding | | |  |
|  | 78 (0.9 ) | 112 (1.6) | |

| **Endpoints from 1 year post MI** | **3 year events, n (%)** | **5 year events, n (%)** | |
| --- | --- | --- | --- |
| All-cause mortality | | |  |
|  | 943 (20.3) | 1,252 (30.6) | |
| CV death or ischaemic/ NOS stroke or MI | | |  |
|  | 745 (16.5) | 936 (23.8) | |
| CV death |  |  | |
|  | 507 (11.5) | 650 (16.9) | |
| Fatal or hospitalised bleeding | | |  |
|  | 211 (4.9) | 292 (8.4) | |
| CALIBER major bleeding | | |  |
|  | 72 (1.7) | 98 (2.7) | |
| Fatal bleeding or intracranial bleeding | | |  |
|  | 39 (0.9 ) | 61 (1.9) | |

**Note**: MI= myocardial infarction, STEMI=ST-elevation myocardial infarction, NSTEMI= non-ST-elevation myocardial infarction, NOS=not otherwise specified, CV=cardiovascular **eTable 4: Patient characteristics at index acute MI discharge and 1 year post-index acute MI in the development (n=12,694) and validation (n=5,613) cohorts**

|  | **Development (n=12,694)** | | **Validation (n=5,613)** | |
| --- | --- | --- | --- | --- |
|  | **At index acute MI discharge** | **1 year post-MI** | **At index acute MI** | **1 year post-MI** |
| Age | 69.1 (12.7) | 70.1 (12.7) | 68.1 (12.8) | 69.1 (12.8) |
| **Behaviours** |  |  |  |  |
| Smoking status |  |  |  |  |
| Ex-smoker | 40.1 | 49.3 | 40.3 | 50.0 |
| Non-smoker | 42 | 36.7 | 38.7 | 34.2 |
| Smoker | 17.9 | 14.0 | 21.1 | 15.8 |
| Alcohol abuse | 9.8 | 10.8 | 14.3 | 15.4 |
| **Cardiovascular diseases** |  |  |  |  |
| Revascularisation (PCI) | 24.8 | 43.5 | 18.8 | 33.0 |
| Heart failure | 18.2 | 23.5 | 21.4 | 28.0 |
| Atrial fibrillation | 14.9 | 18.0 | 14.8 | 17.9 |
| Stroke | 6.0 | 6.9 | 7.0 | 8.1 |
| Peripheral arterial disease | 8.2 | 9.8 | 11 | 13.1 |
| Diabetes |  |  |  |  |
| Type 1 | 1.3 | 1.2 | 0.9 | 0.9 |
| Type 2 | 15.2 | 16.7 | 15.5 | 17 |
| Unspecified | 1.2 | 1.5 | 1.4 | 1.7 |
| Renal disease | 8.7 | 13.6 | 9.5 | 14.8 |
| **Non-cardiovascular diseases** |  |  |  |  |
| COPD | 7.8 | 9.1 | 10.9 | 12.8 |
| Liver disease | 0.3 | 0.4 | 0.5 | 0.5 |
| Non-metastatic cancer | 12.9 | 14.4 | 11.3 | 13.2 |
| Metastatic cancer | 0.8 | 1.0 | 0.7 | 1.2 |
| Dementia | 0.8 | 1.3 | 1.4 | 2.0 |
| Chronic anaemia | 11 | 14.3 | 14 | 17.9 |
| Peptic ulcer | 6.6 | 7.3 | 9.3 | 10.2 |
| Bleeding diatheses and coagulation disorders | 0.8 | 1.1 | 0.9 | 1.1 |
| Hospitalised bleeding | 4.3 | 6.5 | 5.7 | 8.2 |
| **Biomarkers** |  |  |  |  |
| BMI (Continuous) | 28.0 (5.0) | 27.8 (5.1) | 27.9 (5.2) | 27.7 (5.1) |
| BMI (Categorical) |  |  |  |  |
| Underweight | 1.1 | 1.5 | 1.6 | 1.8 |
| Normal | 27.1 | 28.7 | 27.9 | 28.4 |
| Overweight | 41.8 | 41.2 | 42.1 | 42.2 |
| Obese | 30.1 | 28.6 | 28.4 | 27.7 |
| SBP (mmHg) | 145 (16.3) | 133 (18.6) | 144 (17.0) | 132 (18.4) |
| Haemoglobin (g/dL) | 13.9 (1.62) | 13.4 (1.6) | 13.9 (1.61) | 13.3 (1.6) |
| White blood cell count (10^9^/L) | 7.69 (2.19) | 7.60 (2.3) | 7.72 (2.19) | 7.68 (2.3) |
| Total cholesterol (mmol/L) | 5.32 (1.16) | 4.17 (1.0) | 5.38 (1.14) | 4.17 (1.0) |
| HDL cholesterol (mmol/L) | 1.34 (0.40) | 1.28 (0.4) | 1.33 (0.37) | 1.26 (0.4) |
| Creatinine (mol/l) Median (IQR) | 95 (83, 110) | 98 (84, 114) | 97 (85, 113) | 99 (86, 117) |

Note: Categorical prognostic factors are presented as %, continuous prognostic factors are presented as mean (SD) unless stated otherwise

**eFigure 2: Comparison of all-cause mortality, cardiovascular and bleeding events in patents included in the development (n=12,694) and validation (n=5,613) cohorts**


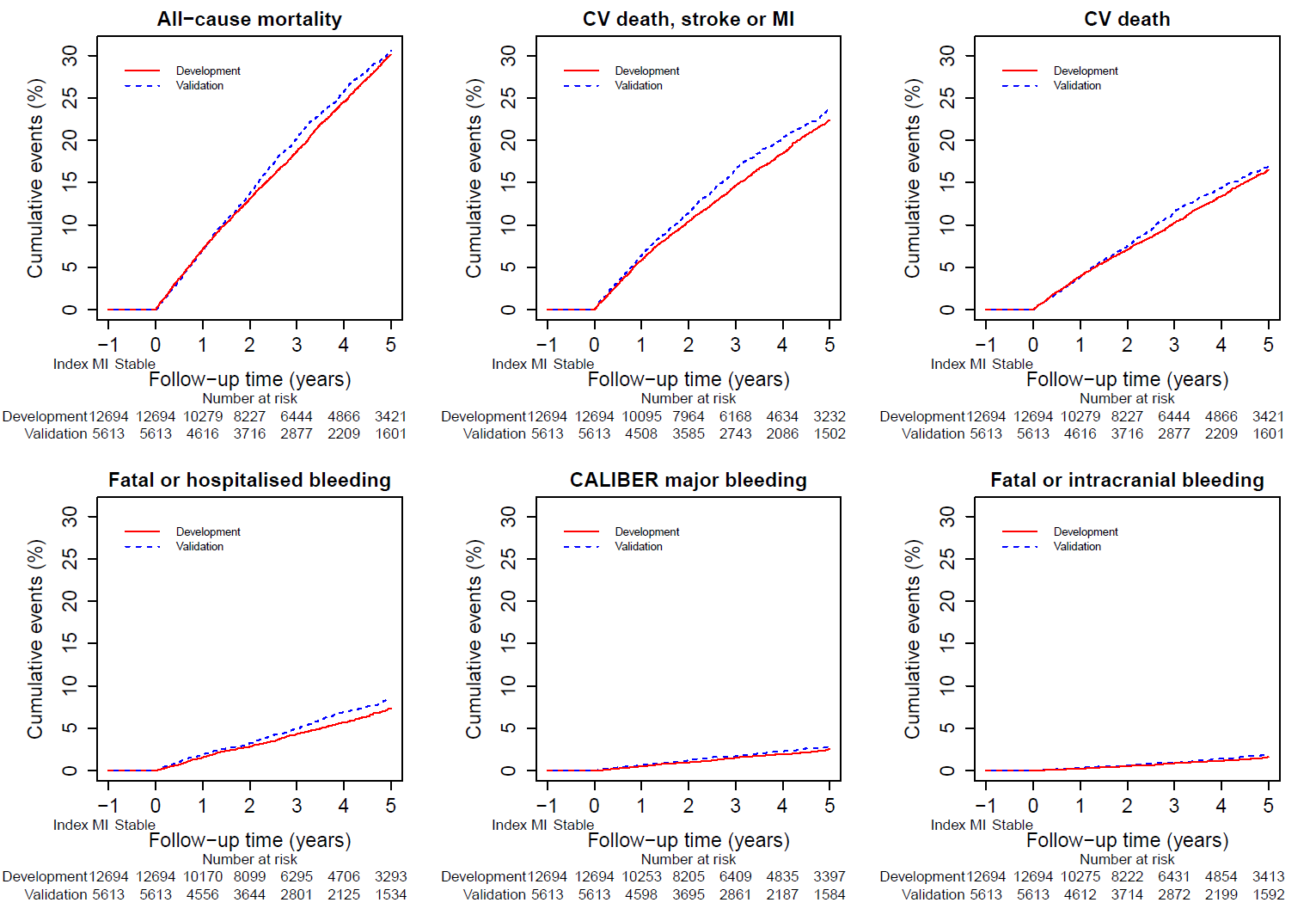


**Note**: CV= cardiovascular, MI=myocardial infarction

**eFigure 3: Univariable effects of prognostic factors on 5 year all-cause mortality, cardiovascular and bleeding endpoints**


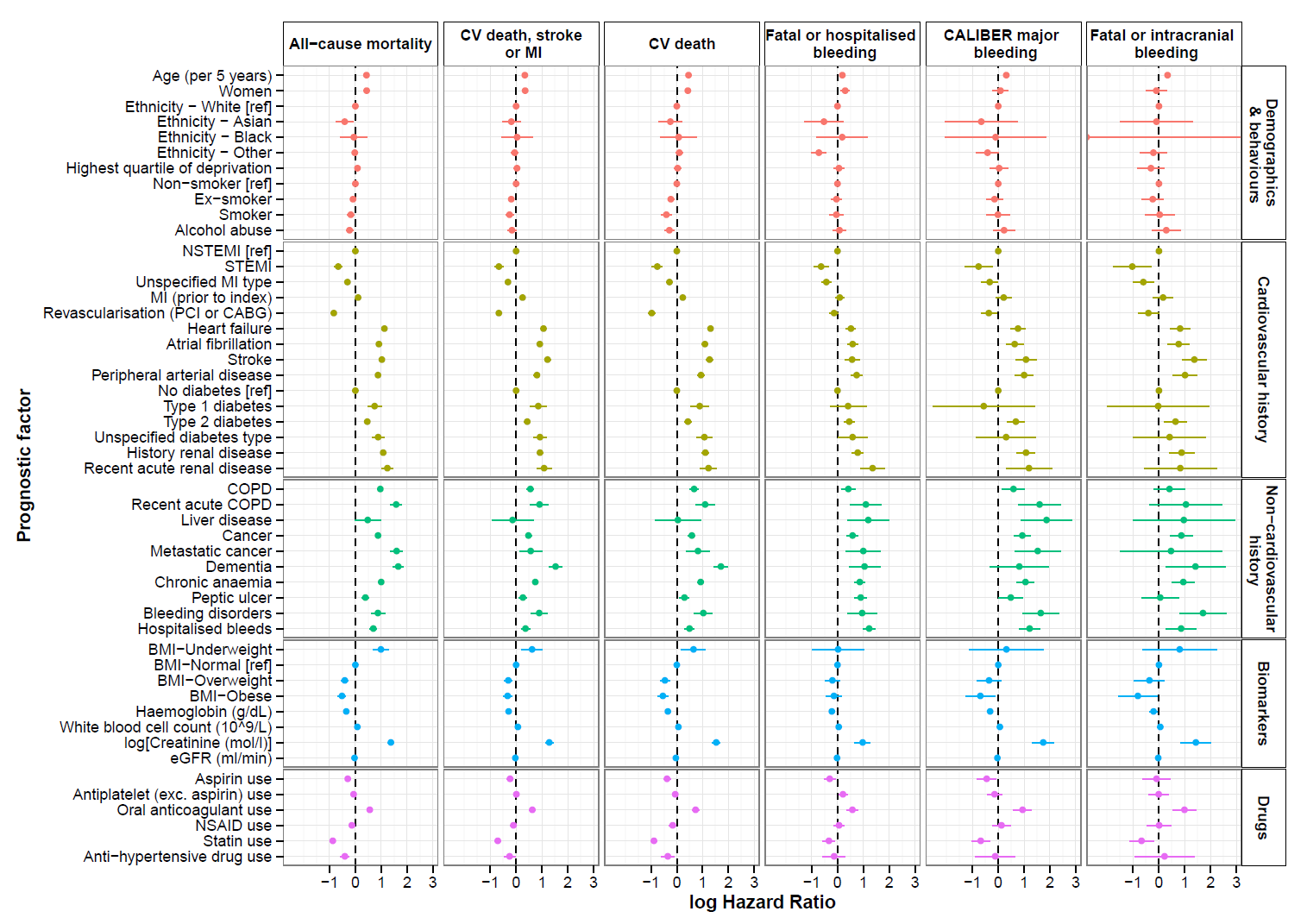


**Note**: CV= cardiovascular, MI= myocardial infarction, NSTEMI= non-ST-elevation myocardial infarction, STEMI= ST-elevation myocardial infarction, COPD= chronic obstructive pulmonary disease, BMI= body mass index, eGFR= estimated glomerular filtration rate, NSAID= non-steroidal anti-inflammatory drugs; (log hazards compared with reference group or per unit increase for continuous prognostic factors and 95% confidence intervals)

**eFigure 4: Univariable proportional hazards assumption checks for the CALIBER major bleeding outcome**


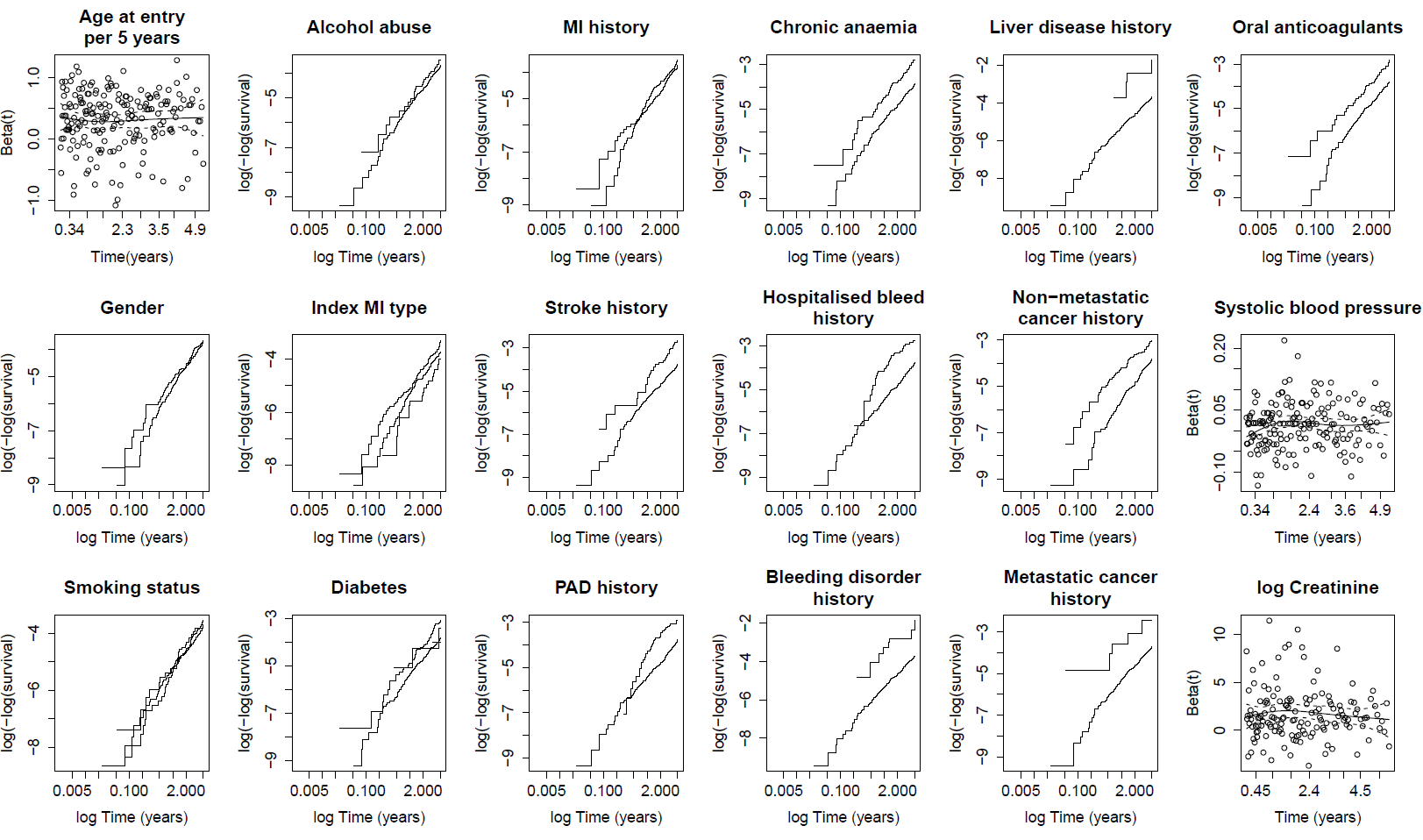


**Note:** For continuous variables, Beta(t) is a time dependent coefficient and should remain constant over time if the proportional hazards assumption have not been violated**.** For categorical variables, proportional hazards have not been violated if the log(-log(survival)) curves remain parallel over time.; MI= myocardial infarction; PAD= peripheral arterial disease

**eTable 5: Multivariable model prognostic hazard ratios and 95% confidence intervals for all-cause mortality, cardiovascular and bleeding endpoints**

|  | **Endpoint** | | | | | |
| --- | --- | --- | --- | --- | --- | --- |
| **Prognostic factor (measured at 1 year post-MI)** | **All-cause mortality** | **Cardiovascular death, stroke or MI** | **Cardiovascular death** | **CALIBER major bleeding** | **Fatal or hospitalised bleeding** | **Fatal bleeding or intracranial bleeding** |
| Age (per 5 years) | 1.38 (1.35, 1.42) | 1.28 (1.24, 1.32) | 1.39 (1.34, 1.45) | 1.3 (1.19, 1.42) | 1.14 (1.09, 1.19) | 1.35 (1.20, 1.52) |
| Women | 0.89 (0.81, 0.98) | 0.89 (0.79, 0.99) | 0.85 (0.75, 0.97) | 0.85 (0.61, 1.2) | 1.15 (0.95, 1.4) | 0.54 (0.34, 0.85) |
| Ethnicity |  |  |  |  |  |  |
| White [ref] | 1 | NA | 1 | NA | 1 | NA |
| Asian | 0.96 (0.68, 1.36) | NA | 1.07 (0.68, 1.68) | NA | 0.68 (0.32, 1.44) | NA |
| Black | 1.19 (0.7, 2.04) | NA | 1.28 (0.63, 2.6) | NA | 1.20 (0.45, 3.22) | NA |
| Other | 1.16 (1.05, 1.28) | NA | 1.26 (1.1, 1.44) | NA | 0.58 (0.43, 0.77) | NA |
| Smoking status |  |  |  |  |  |  |
| Non-smoker [ref] | 1 | 1 | 1 | 1 | 1 | 1 |
| Ex-smoker | 1.02 (0.93, 1.12) | 0.91 (0.82, 1.01) | 0.9 (0.79, 1.02) | 0.88 (0.63, 1.23) | 0.96 (0.79, 1.17) | 0.73 (0.47, 1.14) |
| Smoker | 1.47 (1.28, 1.7) | 1.25 (1.06, 1.48) | 1.22 (0.99, 1.5) | 1.42 (0.87, 2.32) | 1.23 (0.92, 1.64) | 1.44 (0.77, 2.68) |
| Alcohol abuse | 1.24 (1.07, 1.43) | 1.25 (1.07, 1.47) | 1.23 (1.01, 1.5) | 1.69 (1.08, 2.65) | 1.28 (0.97, 1.68) | 1.80 (1.02, 3.19) |
| Index acute MI type |  |  |  |  |  |  |
| NSTEMI [ref] | 1 | 1 | 1 | 1 | 1 | 1 |
| STEMI | 0.81 (0.7, 0.93) | 0.74 (0.63, 0.87) | 0.74 (0.61, 0.91) | 0.67 (0.39, 1.16) | 0.67 (0.5, 0.91) | 0.51 (0.24, 1.06) |
| Unspecified | 0.88 (0.81, 0.97) | 0.78 (0.7, 0.86) | 0.82 (0.73, 0.93) | 0.84 (0.61, 1.15) | 0.78 (0.65, 0.95) | 0.61 (0.40, 0.91) |
| MI (prior to index acute MI) | 1.07 (0.98, 1.16) | 1.23 (1.12, 1.35) | 1.2 (1.08, 1.34) | 1.18 (0.88, 1.59) | 1.04 (0.87, 1.23) | 1.14 (0.77, 1.68) |
| Previous revascularisation | 0.69 (0.63, 0.76) | 0.72 (0.64, 0.8) | 0.61 (0.53, 0.7) | NA | NA | 0.87 (0.56, 1.34) |
| Heart failure | 1.47 (1.35, 1.6) | 1.57 (1.42, 1.74) | 1.74 (1.55, 1.97) | NA | NA | 1.34 (0.88, 2.04) |
| Atrial fibrillation | 1.23 (1.13, 1.34) | 1.33 (1.2, 1.47) | 1.41 (1.26, 1.59) | NA | NA | 0.85 (0.51, 1.42) |
| Stroke | 1.41 (1.26, 1.58) | 1.86 (1.64, 2.11) | 1.73 (1.49, 2) | 1.6 (1.06, 2.43) | 1.13 (0.84, 1.51) | 2.28 (1.38, 3.78) |
| Peripheral arterial disease | 1.48 (1.33, 1.64) | 1.45 (1.28, 1.64) | 1.6 (1.38, 1.84) | 1.55 (1.06, 2.27) | 1.41 (1.11, 1.79) | 1.64 (1.01, 2.69) |
| Diabetes |  |  |  |  |  |  |
| No diabetes | 1 | 1 | 1 | 1 | 1 | 1 |
| Type 1 diabetes | 2.33 (1.75, 3.09) | 2.33 (1.7, 3.2) | 2.44 (1.68, 3.52) | 0.55 (0.08, 4) | 1.48 (0.73, 3) | 0.87 (0.12, 6.40) |
| Type 2 diabetes | 1.22 (1.11, 1.35) | 1.18 (1.05, 1.32) | 1.15 (1, 1.33) | 1.49 (1.06, 2.09) | 1.29 (1.05, 1.59) | 1.48 (0.95, 2.30) |
| Unspecified diabetes | 2.44 (1.93, 3.1) | 2.34 (1.79, 3.07) | 2.89 (2.13, 3.92) | 1.07 (0.33, 3.41) | 1.62 (0.9, 2.89) | 1.31 (0.31, 5.45) |
| Renal disease | 1.23 (1.1, 1.37) | NA | NA | NA | NA | NA |
| COPD | 1.62 (1.45, 1.8) | NA | 1.23 (1.04, 1.44) | NA | NA | NA |
| Liver disease | NA | NA | NA | 3.94 (1.4, 11.11) | NA | NA |
| Non-metastatic cancer | 1.31 (1.19, 1.44) | NA | NA | 1.48 (1.04, 2.12) | 1.26 (1.01, 1.56) | 1.49 (0.95, 2.34) |
| Metastatic cancer | 2.32 (1.81, 2.98) | NA | NA | 2.19 (0.86, 5.58) | NA | NA |
| Dementia | 2.03 (1.64, 2.53) | 2.03 (1.56, 2.64) | 2.16 (1.62, 2.87) | NA | NA | NA |
| Chronic anaemia | 1.13 (1.02, 1.25) | NA | NA | 1.42 (0.99, 2.03) | 1.40 (1.13, 1.73) | 1.42 (0.89, 2.25) |
| Peptic ulcer | NA | NA | 1.02 (0.85, 1.24) | NA | 1.75 (1.37, 2.24) | NA |
| Bleeding diatheses or coagulation disorders | NA | 1.3 (0.95, 1.79) | NA | 2.02 (0.94, 4.32) | NA | 2.49 (0.95, 6.48) |
| Hospitalised bleeding | NA | 0.84 (0.71, 1) | 0.85 (0.69, 1.03) | 1.82 (1.2, 2.78) | 2.01 (1.56, 2.58) | NA |
| BMI |  |  |  |  |  |  |
| Underweight | 1.91 (1.43, 2.53) | 1.6 (1.11, 2.3) | 1.65 (1.09, 2.5) | NA | NA | NA |
| Normal [ref] | 1 | 1 | 1 | NA | NA | NA |
| Overweight | 0.93 (0.82, 1.05) | 0.95 (0.84, 1.08) | 0.92 (0.78, 1.08) | NA | NA | NA |
| Obese | 1 (0.88, 1.15) | 1.05 (0.91, 1.22) | 1.02 (0.85, 1.24) | NA | NA | NA |
| White blood cell count (10^9^/L) | 1.06 (1.03, 1.08) | 1.05 (1.02, 1.07) | 1.05 (1.02, 1.08) | NA | NA | NA |
| Haemoglobin (g/dL) | 0.88 (0.85, 0.92) | 0.91 (0.88, 0.95) | 0.89 (0.85, 0.93) | NA | NA | NA |
| log Creatinine (μmol/l) | 1.35 (1.15, 1.59) | 1.49 (1.25, 1.77) | 1.72 (1.41, 2.1) | 2.15 (1.29, 3.57) | 1.44 (1.05, 1.97) | NA |
| Cholesterol ratio (HDL:total) | 1.58 (0.92, 2.71) | 1.46 (0.82, 2.59) | 1.56 (0.77, 3.15) | NA | NA | NA |
| Antiplatelet | NA | NA | NA | NA | 1.23 (1.03, 1.47) | NA |
| Oral anticoagulant | NA | NA | NA | 1.74 (1.19, 2.52) | 1.49 (1.17, 1.9) | 1.89 (1.08, 3.33) |

**Note**: All models are adjusted for systolic blood pressure using restricted cubic splines. Functions are shown in **eTable 5**; MI=myocardial infarction, NSTEMI= non-ST-elevation myocardial infarction, STEMI= ST-elevation myocardial infarction, COPD= chronic obstructive pulmonary disease, HDL= high-density lipoprotein

**eFigure 5: U-shaped association of systolic blood pressure and 5 year cardiovascular death, stroke or MI events [n=12,694, events=1,913]**


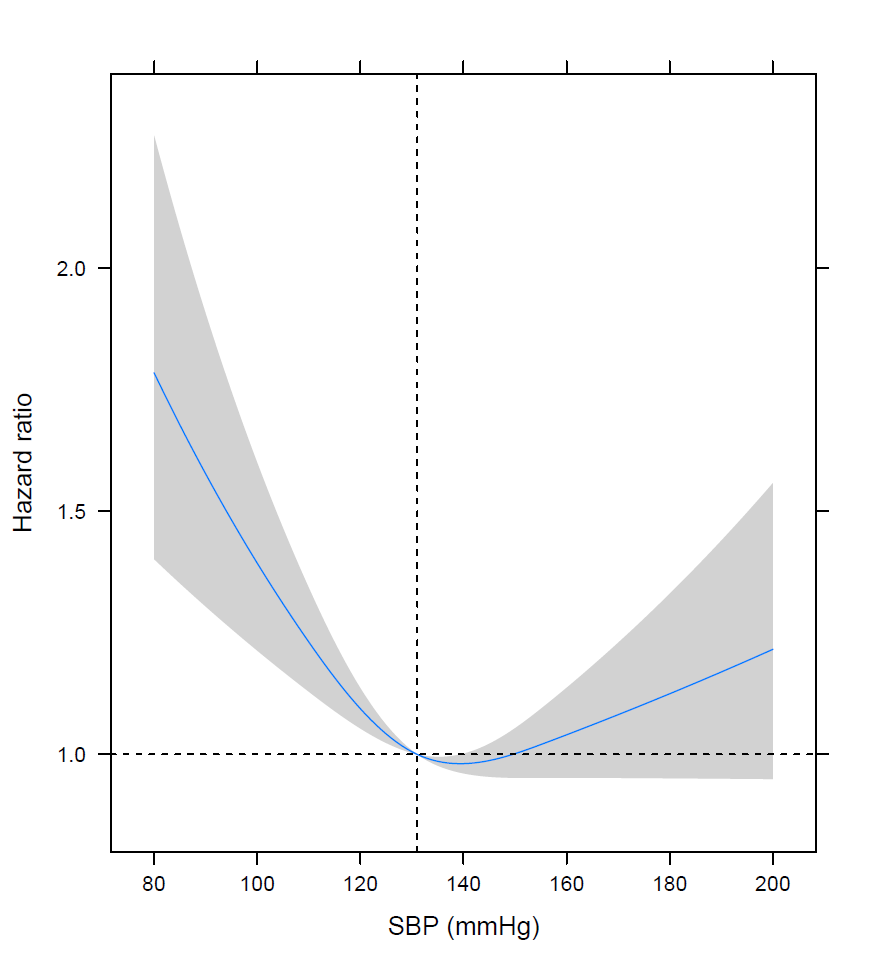


**Note:** MI= myocardial infarction; SBP= systolic blood pressure

**eTable 6: Linear predictor functions for systolic blood pressure in the multivariable models**

| **Endpoint** | **Function** |
| --- | --- |
| All-cause mortality | 0.011 x SBP - 3.566 x 10^-6^ x max(SBP-110,0)^3^ + 6.562 x 10^-6^ x max(SBP-131,0)^3^ - 2.996 x 10^-6^ x max(SBP-156,0)^3^ |
| Cardiovascular death, stroke or MI | 0.012 x SBP - 5.619 x 10^-6^ x max(SBP-110,0)^3^ + 1.034 x 10^-5^ x max(SBP-131,0)^3^ -4.720 x 10^-6^ x max(SBP-156,0)^3^ |
| Cardiovascular death | 0.015 x SBP - 6.383 x 10^-6^ x max(SBP-110,0)^3^ + 1.174 x 10^-5^ x max(SBP-131,0)^3^ -5.362 x 10^-6^ x max(SBP-156,0)^3^ |
| CALIBER major bleeding | 0.006* SBP -5.722 x 10^-6^ x max(SBP -110,0)^3^+ 1.0529 x 10^-5^ x max(SBP -131,0)^3^-4.807 x 10^-6^ x max(SBP -156,0)^3^ |
| Fatal or hospitalised bleeding | 0.010 x SBP - 6.231 x 10^-6^ x max(SBP-110,0)^3^ + 1.146 x 10^-5^ x max(SBP-131,0)^3^ - 5.234 x 10^-6^ x max(SBP-156,0)^3^ |
| Fatal bleeding or intracranial bleeding | 0.002 x SBP -5.176 x 10^-6^ x max(SBP-110,0)^3^+9.524 x 10^-6^ x max(SBP-131,0)^3^-4.348 x 10^-6^ x max(SBP-156,0)^3^ |

**Note**: Systolic blood pressure was modelled using restricted cubic splines with 3 knots in the multivariable models. The functions in this table described the estimated relationship between systolic blood pressure and the studied endpoints

**eTable 7:** **Percentages of patients in the 4 risk groups from applying the 16%, 50% and 84% cut-points of the linear predictors calculated with the development cohort for each endpoint**

|  |  | **Risk Group** | | | |
| --- | --- | --- | --- | --- | --- |
| **Endpoint** | **Cohort** | **Highest** | **High** | **Low** | **Lowest** |
| CV death stroke MI | Development | 16.0 | 34.0 | 34.0 | 16.0 |
|  | Validation | 17.7 | 34.2 | 33.6 | 14.5 |
| Fatal or intracranial bleeding | Development | 16.0 | 34.0 | 34.0 | 16.0 |
|  | Validation | 16.1 | 33.7 | 34.9 | 15.4 |
| CALIBER major bleeding | Development | 16.0 | 34.0 | 34.0 | 16.0 |
|  | Validation | 16.7 | 33.5 | 34.6 | 15.3 |
| Hospitalised bleeding | Development | 16.0 | 34.0 | 34.0 | 16.0 |
|  | Validation | 18.5 | 33.5 | 33.2 | 14.8 |

Note: Development cohort (n=12,694), Validation cohort (n=5,613)

**eFigure 6: Overlap of 3 year predicted risks based on multivariable models in those with and without categorical risk factors: age≥ 65, diabetes, history of MI and renal disease (used to define high risk in the PEGASUS-TIMI 54 trial)**


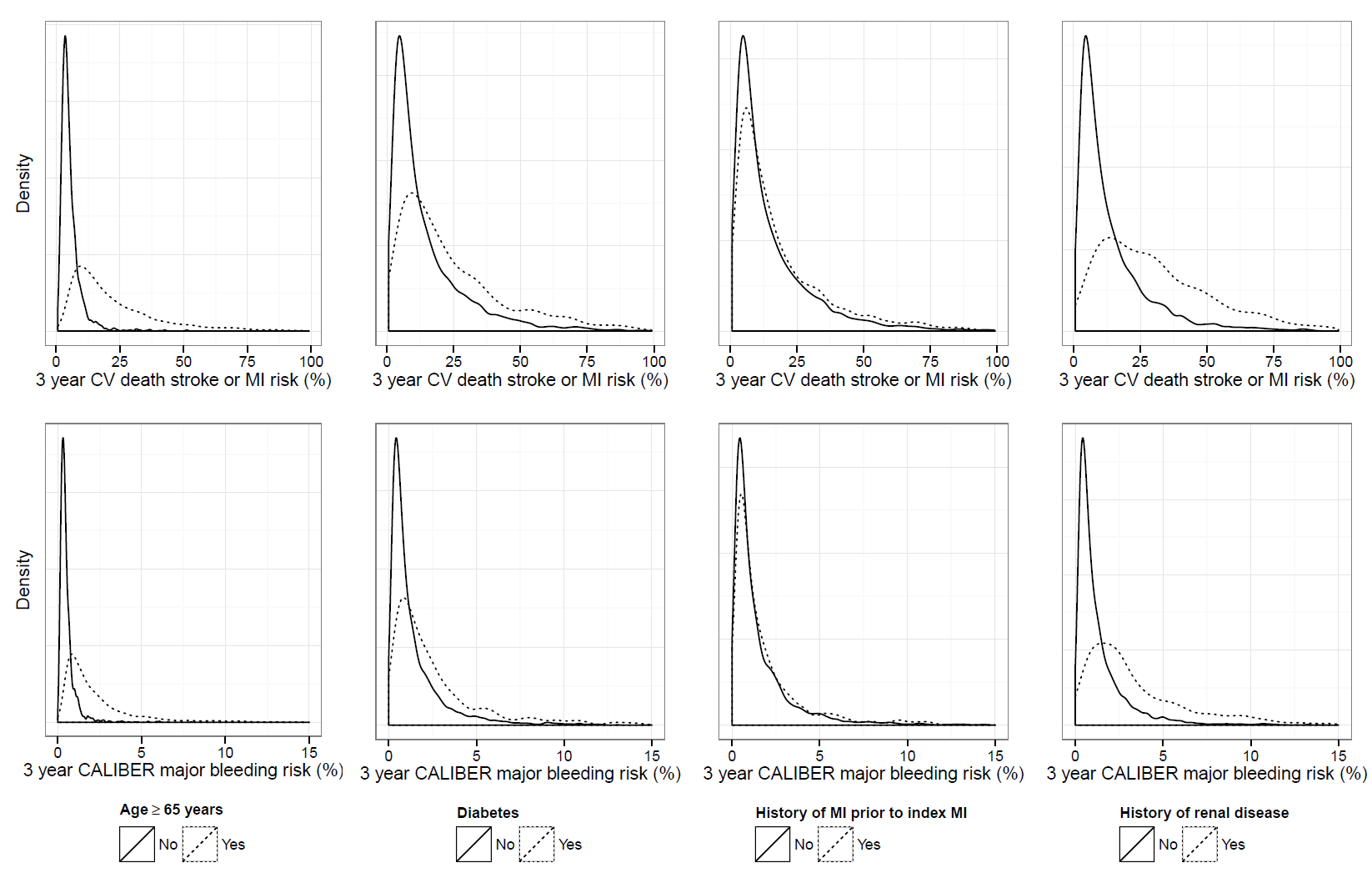


Note: Each panel shows the distribution of predicted 3-year CV or bleeding risks in patients with and without 4 binary indicators of high CV risk (Age, diabetes, MI history, renal disease). The dotted curve is the distribution of risk for patients with the high risk factor and the solid curve is the distribution of risk without the high risk factor. We demonstrate that these ‘high risk’ factors alone are insufficient to separate patients who are truly at higher and lower risks. CV= cardiovascular; MI= myocardial infarction
